# Anaphylactic degranulation by mast cells requires the mobilization of inflammasome components

**DOI:** 10.1038/s41590-024-01788-y

**Published:** 2024-03-14

**Authors:** Andrea Mencarelli, Pradeep Bist, Hae Woong Choi, Hanif Javanmard Khameneh, Alessandra Mortellaro, Soman N. Abraham

**Affiliations:** 1 Program in Emerging Infectious Diseases, Duke-National University of Singapore, Singapore, Singapore; 2 Department of Pathology, Duke University Medical Center, Durham, NC, USA; 3 Singapore Immunology Network (SIgN), Agency for Science and Research (A*Star), Singapore, Singapore; 4 San Raffaele Telethon Institute for Gene Therapy (SR-Tiget), IRCCS San Raffaele Scientific Institute, Milan, Italy; 5 Department of Immunology, Duke University Medical Center, Durham, NC, USA; 6 Department of Molecular Genetics & Microbiology, Duke University Medical Center, Durham, NC, USA; 7 Present address: Shanghai Immune Therapy Institute, Renji Hospital, Shanghai Jiao Tong University School of Medicine, Shanghai, China; 8 Present address: Università della Svizzera italiana (USI), Faculty of Biomedical Sciences, Institute for Research in Biomedicine (IRB), Bellinzona, Switzerland

## Abstract

The inflammasome components NLRP3 and ASC are cytosolic proteins, which upon sensing endotoxins or danger cues, form multimeric complexes to process interleukin (IL)-1β for secretion. Here we found that antigen (Ag)-triggered degranulation of IgE-sensitized mast cells (MCs) was mediated by NLRP3 and ASC. IgE–Ag stimulated NEK7 and Pyk2 kinases in MCs to induce the deposition of NLRP3 and ASC on granules and form a distinct protein complex (granulosome) that chaperoned the granules to the cell surface. MCs deficient in NLRP3 or ASC did not form granulosomes, degranulated poorly in vitro and did not evoke systemic anaphylaxis in mice. IgE–Ag-triggered anaphylaxis was prevented by an NLRP3 inhibitor. In endotoxin-primed MCs, pro-IL-1β was rapidly packaged into granules after IgE–Ag stimulation and processed within granule remnants by proteases after degranulation, causing lethal anaphylaxis in mice. During IgE–Ag-mediated degranulation of endotoxin-primed MCs, granulosomes promoted degranulation, combined with exteriorization and processing of IL-1β, resulting in severe inflammation.

The inflammasome is a large intracellular multicomplex formed in immune cells consisting of a cytosolic sensor protein, NLRP3, an adaptor protein, ASC, and a downstream effector protein, pro-caspase-1 (ref. 1), which serves to promote inflammation. Upon sensing pathogen-associated molecular patterns such as lipopolysaccharide (LPS), microbial toxins such as nigericin or danger-associated molecular patterns such as ATP, cytosolic inflammasome complexes activate caspase-1 to rapidly process proinflammatory cytokines such as IL-1β, which, when released, trigger immune reactions ranging from recruitment and activation of various immune cells to the production of pyrogens^2,3^. The formation of the inflammasome complex is initiated by a variety of intracellular cues, including Ca^2+^ influx^4^. Early inflammasome assembly-related cellular events include the dimerization of the immune sensor NLRP3 (ref. 5) by its regulator, NEK7, and the phosphorylation of the adaptor protein ASC into an oligomerization-competent state by a protein tyrosine kinase Pyk2 (ref. 6). Although inflammasome constituents are present in different cell types and are associated with various inflammatory, metabolic and neurogenerative diseases^7–11^, they have so far been mainly linked to secretion of IL-1β or related proinflammatory cytokines^2,3^.

Most studies on inflammasome formation and function have focused on resident phagocytes^12^. Mast cells (MCs) are tissue-resident secretory cells that are heavily granulated and localized around blood vessels and at the host–environment interface^13^. Upon activation, MCs degranulate, triggering a cascade of immune reactions through the release of granule-associated and de novo synthesized proinflammatory mediators^14^. Because mediators that are prestored in granules are released in bulk immediately upon activation, their impact on immune responses is both rapid and profound. Upon priming with LPS and subsequent stimulation by nigericin, MCs release appreciable amounts of IL-1β in a manner dependent on the canonical NLRP3 inflammasome. The constitutive release of IL-1β seen in the skin of individuals with the cryopyrin-associated periodic syndrome is largely associated with MCs^15^.

MCs are best known for triggering life-threatening IgE-mediated anaphylaxis upon contact with various blood-borne allergens^16^. IgE-FcεRI-mediated MC activation leads to a large increase in cytosolic Ca^2+^ levels^17^, while the final steps of MC degranulation involve interactions of SNARE proteins localized on opposing membranes to mediate fusion of membrane-bound fused granules to the plasma membrane and release of granules^18^. How cytoplasmic MC granules are mobilized and trafficked to the plasma membrane following activation remains relatively unclear. Here, we found that, following IgE-mediated activation, the inflammasome components NLRP3 and ASC form multimeric complexes with MC granules to chaperone these granules along organized microtubules (MTs) to the cell surface for exteriorization.

## Results

### Inflammasome components mediate MC degranulation

**Extended Data Fig. 1 |:**
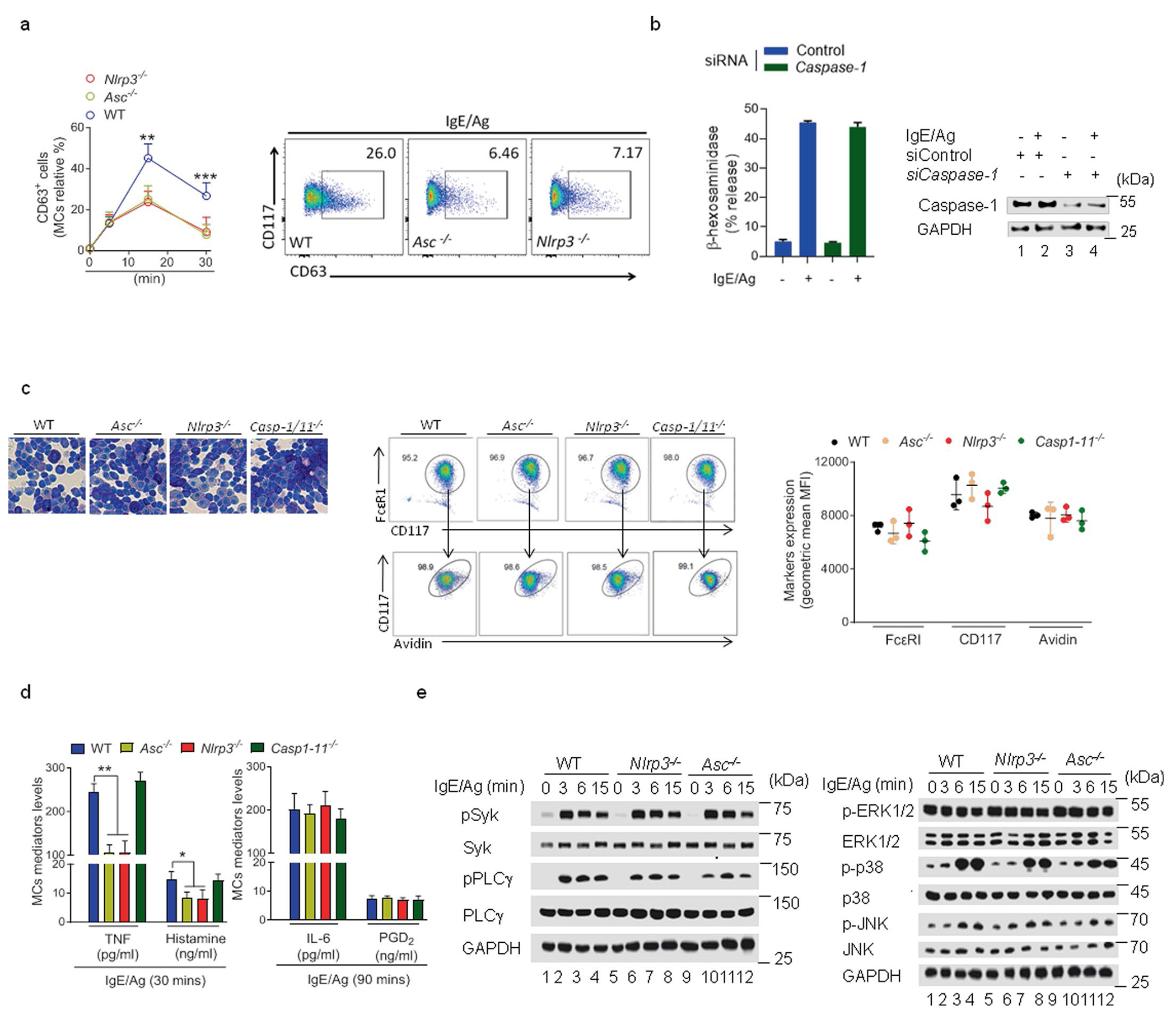
*Nlrp3*^−/−^ and *Asc*^−/−^ BMMCs exhibit similar early signaling events as WT. **a**, The impaired FcεRI-mediated degranulation observed in MCs ablated of *Asc* and *Nlrp3* was also confirmed by flow cytometric analysis of the externalization of the granule membrane marker CD63 after IgE-Ag stimulation. **b** β-hexosaminidase release from IgE-Ag stimulated WT BMMCs silenced for siRNA targeting Control or *Caspase-1*. Western blot analysis of Caspase-1 protein levels. **c**, The BMMCs were subjected to toluidine blue staining which stains mature MC granule. Middle, flow cytometry analysis of BMMCs obtained from WT, *Asc*^−/−^ *Nlrp3*^−/−^ or *Caspase^1/11−/−^* mice showing that all BMMCs were similarly matured. The maturation markers employed were FcεRI, CD117, and avidin, respectively. **d**, Compared to WT BMMCs, *Asc*^−/−^, *Caspase-^1/11−/−^* and *Nlrp3*^−/−^ BMMCs exhibit significantly reduced release of granule associated mediator’s TNF-α and histamine following IgE-Ag-mediated stimulation. Compared to WT BMMCs, *Asc*^−/−^ and *Nlrp3*^−/−^ BMMCs exhibited similar levels of secretion of *de novo* synthesized mediators PGD_2_ and IL-6. **e**, Western blots showing IgE-sensitized WT, *Nlrp3*^*−/−*^ or *Asc*^−/−^ BMMCs stimulated with Ag at different time points. The data are shown in a as mean ± s.d of two independent experiments, performed in triplicate, **P < 0.01, ***P < 0.005 (Ordinary one-way ANOVA multiple comparisons procedure), WT vs. *Asc*^−/−^ and *Nlrp3*^−/−^ BMMCs, for each time point indicated. Data are shown in b are mean ± s.e.m of one representative experiment, of three independent experiments with similar results, performed in triplicate; The images shown in c, are the representatives of four independent experiments, with similar results. The data are shown in c left, as mean ± s.d. of four independent experiments. The data are shown in d as mean ± s.d of two independent experiments, performed in duplicate or triplicate, **P < 0.01 (Ordinary one-way ANOVA multiple comparisons procedure), WT vs. *Asc*^−/−^ and *Nlrp3*^−/−^ BMMCs as indicated. Data shown in e are representative of three independent experiments.

**Fig. 1 |:**
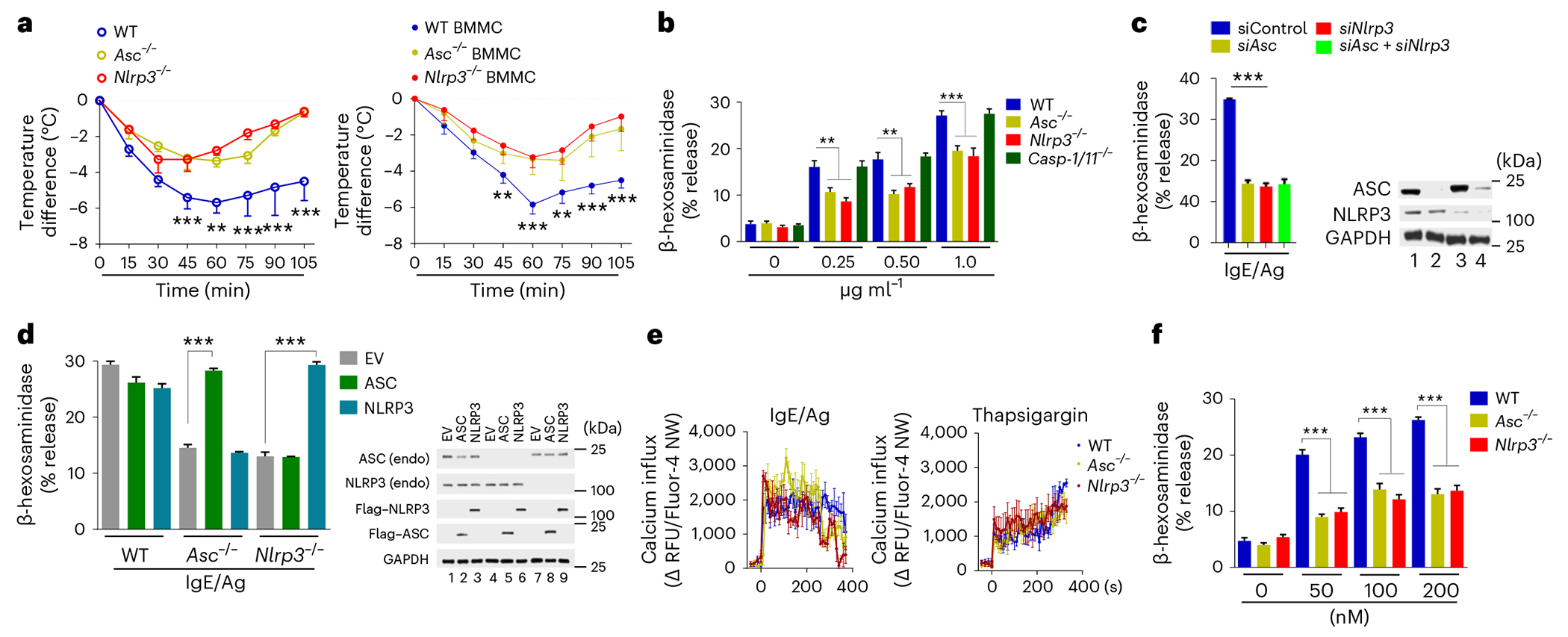
Inflammasome components mediate MC degranulation following IgE–Ag stimulation. **a**, Body temperature changes in wild-type (WT), *Asc*^−/−^ and *Nlrp3*^−/−^ mice and in Kit*^W-sh/W-sh^* mice repleted with BMMCs from WT, *Asc*^−/−^ or *Nlrp3*^−/−^ mice sensitized with TNP-specific IgE antibody and injected i.v. with TNP-OVA and monitored for 105 min. **b**, β-hexosaminidase release from DNP-specific IgE-sensitized WT, *Asc*^−/−^, *Nlrp3*^−/−^ and *Casp-1/Casp-11*^−/−^ BMMCs after exposure to increasing doses of BSA-DNP. **c**, β-hexosaminidase release from IgE-sensitized and BSA-DNP stimulated RBL-2H3 MCs in which the expression of *Asc*, *Nlrp3* or both was silenced with siRNA and immunoblot analysis of NLRP3 and ASC expression in each condition, with GAPDH as a loading control.**d**, β-hexosaminidase release from IgE-sensitized, BSA-DNP-stimulated *Asc*^−/−^ or *Nlrp3*^−/−^ BMMCs transduced with plasmids encoding mouse ASC, NLRP3 or empty vector (EV) and an immunoblot of endogenous NLRP3 and ASC and Flag–NLRP3 and Flag–ASC in cell lysates of each experimental condition. **e**, Measurement of intracellular Ca^2+^ concentrations using Fluo-4 NW assay kit in WT, *Asc*^−/−^ and *Nlrp3*^−/−^ BMMCs after IgE–Ag or thapsigargin exposure. **f**, β-hexosaminidase release from WT, *Asc*^−/−^ and *Nlrp3*^−/−^ BMMCs following exposure to thapsigargin (0, 50, 100 or 200 nM). Data presented in **a** (left, *n* = 5 mice per group; right, *n* = 7 mice per group) are the mean ± s.d. of **P < 0.01, ***P < 0.005 (ordinary one-way analysis of variance (ANOVA) multiple-comparisons procedure), WT versus *Asc*^−/−^ and *Nlrp3*^−/−^ groups, for each time point indicated. Data shown in **c** are the mean ± s.e.m. of one representative experiment, of three independent experiments with similar results, performed in triplicate; ***P < 0.005 (ordinary one-way ANOVA multiple-comparisons procedure) versus siControl group. Data presented in **d** illustrate the mean ± s.e.m. of one representative experiment, of three independent experiments with similar results, performed in triplicate; ***P < 0.005 (unpaired, two-tailed Student’s *t*-test) as indicated. Data presented in **e** illustrate the mean ± s.d. of one representative experiment, of three independent experiments with similar results, performed in triplicate. Data shown in **b** and **f** are the mean ± s.d. of three or four independent experiments, performed in duplicate or triplicate, **P < 0.01 and ***P < 0.001 WT versus *Asc*^−/−^ and *Nlrp3*^−/−^ BMMCs, ANOVA/Tukey multiple-comparisons procedure. ΔRFU, change in relative fluorescence units.

Because single-cell transcriptomic analysis reported that *Nlrp3* and *Asc*, but not *Caspase-1* mRNA, were constitutively expressed in mouse MCs at the steady state^19^, we investigated the role of each inflammasome component in IgE–Ag-mediated MC activation. For these studies, we used trinitrophenyl hapten conjugated to ovalbumin (hereafter referred to as Ag) and mouse trinitrophenyl (TNP)-specific IgE antibodies (hereafter referred to as IgE). IgE-sensitized *Nlrp3*^−/−^ mice or *Asc*^−/−^ mice challenged with Ag exhibited limited anaphylactic responses compared to wild-type mice, which experienced a significant drop in core body temperature (Fig. 1a). To investigate if the limited anaphylactic responses observed in *Nlrp3*^−/−^ and *Asc*^−/−^ mice was specifically attributable to deficiencies in the inflammasome component within MCs, we cultured bone marrow-derived mast cells (BMMCs) from *Nlrp3*^−/−^, *Asc*^−/−^ or wild-type mice and then transferred them separately into MC-deficient *Kit^W-sh/W-sh^* mice^20^. Whereas IgE-sensitized *Kit^W-sh/W-sh^* mice repleted with wild-type BMMCs exhibited a powerful anaphylaxis response when intravenously (i.v.) administered with Ag, *Kit^W-sh/W-sh^* mice repleted with *Asc*^−/−^ or *Nlrp3*^−/−^ BMMCs exhibited minimal anaphylactic reactions (Fig. 1a), suggesting that ASC and NLRP3 were required in MCs for anaphylactic reactions. We also assessed IgE-mediated MC degranulation responses to increasing concentrations of Ag by wild-type, *Asc*^−/−^, *Nlrp3*^−/−^ and *Casp-1/Casp-11*^−/−^ BMMCs in vitro by assessing the amounts of the granule component β-hexosaminidase in the extracellular medium. Regardless of the Ag doses tested, *Asc*^−/−^ and *Nlrp3*^−/−^, but not *Casp-1/Casp-11*^−/−^ BMMCs, exhibited reduced β-hexosaminidase release compared to wild-type BMMCs (Fig. 1b). In an independent assay for MC degranulation where we quantified expression of CD63, a marker expressed on intracellular granule membranes that becomes exposed on MC surfaces when MC granule contents are released, *Asc*^−/−^ and *Nlrp3*^−/−^ MCs exhibited reduced surface expression of CD63 compared to wild-type MCs (Extended Data Fig. 1a).

Next, IgE–Ag stimulation of rat RBL-2H3 MCs^21^ in which *ASC* and *NLRP3* were knocked down (KD) separately or simultaneously using short interfering RNA (siRNA) resulted in reduced release of β-hexosaminidase in the *ASC* and *NLRP3* single or double KD RBL-2H3 cells compared to control siRNA-transfected RBL-2H3 cells (Fig. 1c). The impairment of β-hexosaminidase release was similar in ASC KD, NLRP3 KD and the double KD RBL-2H3 cells (Fig. 1c), suggesting that NLRP3 and ASC contributed to MC degranulation in the same signaling circuitry. siRNA KD of *Casp-1* in RBL-2H3 cells degranulated to the same degree as control siRNA-transfected RBL-2H3 cells following IgE–Ag stimulation (Extended Data Fig. 1b), indicating that caspase-1 was dispensable for MC degranulation. Exogenous expression of NLRP3 or ASC rescued the release of β-hexosaminidase in *Nlrp3*^−/−^ or *Asc*^−/−^ BMMCs, respectively, to levels seen in wild-type BMMCs upon IgE–Ag stimulation (Fig. 1d). However, overexpression of NLRP3 in *Asc*^−/−^ or wild-type BMMCs did not have a significant impact on MC β-hexosaminidase release (Fig. 1d). Similarly, overexpression of ASC in *Nlrp3*^−/−^ or wild-type BMMCs did not affect MC degranulation (Fig. 1d), suggesting they perform distinct roles. Immunoblotting confirmed the expression of NLRP3 or ASC from the transfected plasmids in *Nlrp3*^−/−^, *Asc*^−/−^ or wild-type BMMCs (Fig. 1d). *Nlrp3*^−/−^ or *Asc*^−/−^ BMMCs appeared fully granulated upon toluidine staining and expressed avidin-FITC, a specific probe for granule heparin, IgE receptor (FcεRI) and CD117 (c-kit), which are markers of mature MCs, at similar levels to wild-type BMMCs (Extended Data Fig. 1c), suggesting that neither *Nlrp3* nor *Asc* affected MC differentiation and growth in culture. Upon IgE–Ag stimulation, MCs release prestored (tumor necrosis factor (TNF) and histamines) mediators through degranulation and de novo synthesized (IL-6 and PGD_2_) inflammatory mediators^22^. TNF and histamine released by *Nlrp3*^−/−^ or *Asc*^−/−^ MCs following IgE–Ag stimulation were reduced compared to wild-type and *Casp-1/Casp-11*^−/−^ BMMCs (Extended Data Fig. 1d). However, secretion of IL-6 and PGD_2_ was similar in *Nlrp3*^−/−^, *Asc*^−/−^, *Casp-1/11*^−/−^ and wild-type BMMCs (Extended Data Fig. 1d), indicating that FcεRI-mediated signals associated with de novo synthesis of mediators were independent of *Nlrp3* or *Asc*.

We next examined the molecular signaling events downstream of IgE–Ag stimulation in *Nlrp3*^−/−^ and *Asc*^−/−^ BMMCs. Phosphorylation of Syk and PLCγ (Extended Data Fig. 1e) and phosphorylation of the MAPKs kinases ERK1/ERK2, p38 and JNK were comparable in *Nlrp3*^−/−^, *Asc*^−/−^ and wild-type BMMCs (Extended Data Fig. 1e), indicating that NLRP3 and ASC were not involved in these signaling events. An increase in intracellular Ca^2+^ is a critical milepost in IgE–Ag signaling in MCs following FcεRI-initiated membrane signaling events. The Ca^2+^ response was comparable in *Nlrp3*^−/−^, *Asc*^−/−^ BMMCs and wild-type BMMCs (Fig. 1e), suggesting NLRP3 and ASC acted downstream of this response. Thapsigargin, a trigger of cytosolic Ca^2+^ that bypasses the early membrane signaling events^23^, induced comparable Ca^2+^ responses in *Nlrp3*^−/−^, *Asc*^−/−^ and wild-type BMMCs (Fig. 1e) but lower degranulation in *Nlrp3*^−/−^ and *Asc*^−/−^ compared to wild-type BMMCs (Fig. 1f), suggesting that the degranulation defect in *Nlrp3*^−/−^ and *Asc*^−/−^ BMMCs was downstream of the Ca^2+^ response. Thus, NLRP3 and ASC were necessary for IgE–Ag-mediated MC degranulation downstream of FcεRI-initiated membrane signaling events.

### NLRP3 and ASC form complexes with CD63 on MC granules

**Extended Data Fig. 2 |:**
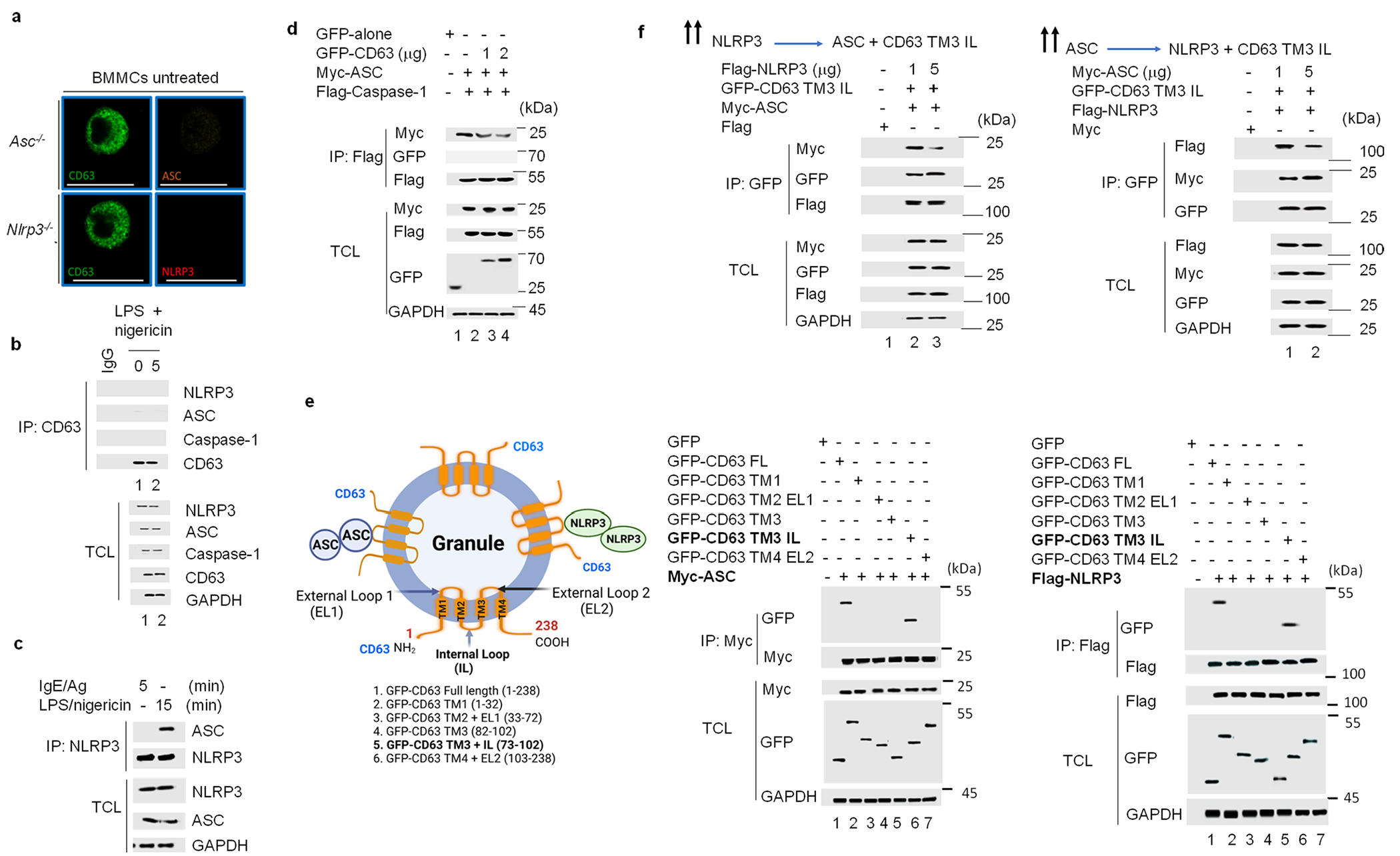
The specificity of NLRP3 and ASC for MC granules CD63. **a**, Immunofluorescence images of *Asc*^−/−^ and *Nlrp3*^−/−^ BMMCs co-stained with CD63 (green) and NLRP3 (red) or ASC (orange), respectively. Scale bar: 20 μm. **b**, Endogenous CD63 was immunoprecipitated from untreated or LPS/ nigericin stimulated WT BMMCs. The association between CD63 and NLRP3, ASC, and Caspase-1 in different conditions was examined by Western blotting of the IP fractions. The proteins of interests in the total cell lysate (TCL) were also depicted to indicate that a similar level of CD63, NLRP3, ASC, and Caspase-1 protein present in each fraction. **c**, WT BMMCs were stimulated either with IgE-Ag or LPS/nigericin for the indicated time intervals, and the lysates were subjected to immunoprecipitation using NLRP3 antibody followed by immunoblotting with ASC, and NLRP3 antibodies. TCL was loaded as an input control. **d**, Myc-tagged ASC and Flag-tagged Caspase-1 with varying amounts of GFP-CD63 was overexpressed in HEK293T cells, and immunoprecipitated with Flag antibody revealing the binding of Caspase-1 with ASC by Western blotting of the IP fraction. The proteins of interests in the total cell lysate (TCL) was also depicted to indicate a similar level of CD63, ASC, and Caspase-1 protein present in each fraction. **e**, A diagrammatic representation of the spatial orientation of CD63 bound to either NLRP3 or ASC on granule membrane. Bottom, full length and its truncated forms of GFP-CD63 (1-6) used for immunoprecipitation assay with either Myc-tagged ASC or Flag-tagged NLRP3 in HEK293T cells. **f**, Western blots showing immunoprecipitation of GFP-tagged CD63 TM3 domain with varying amounts of ASC or NLRP3 in HEK293T cells. Two dark arrows indicate the varying concentration of NLRP3 or ASC expression plasmids. Immunofluorescence image data shown in **a** is representative of three independent experiments. The data shown in **b** is representative of two independent experiments. The data shown in **c** to **e** are representative of more than three independent experiments.

**Fig. 2 |:**
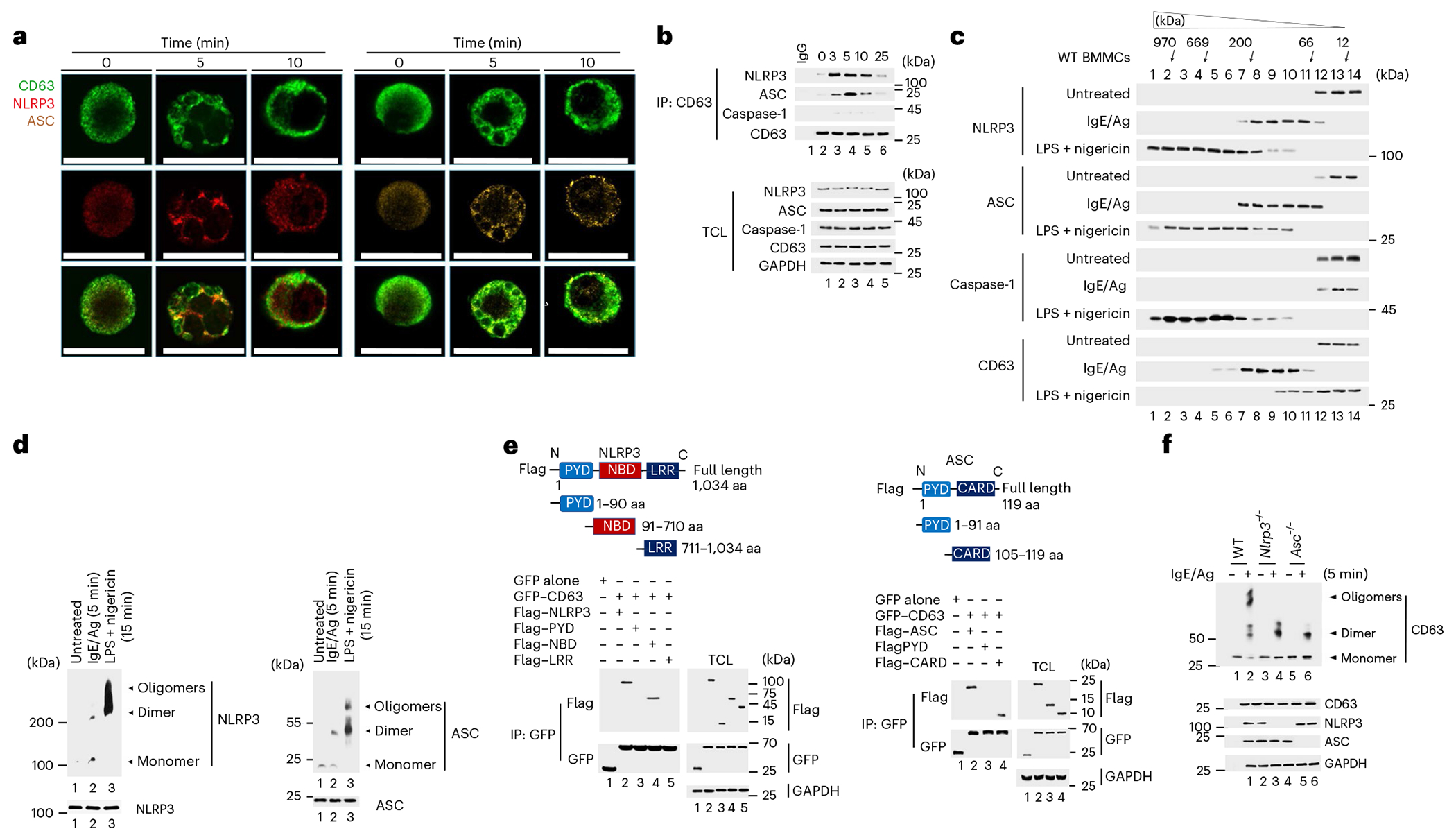
NLRP3 and ASC form a distinct complex with CD63 on MC granules. **a**, Immunofluorescence staining of WT BMMCs with CD63 (green), NLRP3 (red, left) or CD63 (green) and ASC (orange, right) at 0, 5 and 10 min following IgE–Ag stimulation. Scale bars, 20 μm. **b**, Immunoblotting of immunoprecipitated endogenous CD63 with endogenous NLRP3, ASC and caspase-1 in the immunoprecipitation (IP) fractions from untreated or IgE–Ag stimulated WT BMMCs. IgG antibody from the corresponding rabbit or mouse species was used as a control. Protein of interests in the total cell lysates (TCL) are depicted in each fraction. **c**, Immunoblot analyses of NLRP3, ASC, caspase-1 and CD63 in cell fractions from untreated, IgE–Ag-stimulated or LPS/nigericin-stimulated WT BMMCs obtained by SEC. **d**, Protein immunoblot analysis of cross-linked NLRP3 and ASC from untreated, IgE–Ag-stimulated or LPS/nigericin-stimulated WT BMMCs. **e**, Schematics showing various tagged full and truncated domains of NLRP3 and ASC (top) and immunoblotting with Flag-specific antibodies in GFP-CD63 immunoprecipitates from HEK293T cells overexpressing Flag–NLRP3, Flag–PYD, Flag–NBD, Flag–LRR and Flag–ASC (bottom). **f**, Immunoblots using CD63-specific antibody cross-linked in WT, *Nlrp3*^−/−^ and *Asc*^−/−^ BMMCs activated with IgE–Ag. The immunofluorescence images shown in **a** are representative of three or more independent experiments. Data shown in **b**–**f** are representative of three or more independent experiments. aa, amino acid.

Next, we used confocal microscopy to investigate a physical association between MC granules with either NLRP3 or ASC. At steady state, CD63^+^ MC granules were weakly associated with both NLRP3 and ASC (Fig. 2a). Within 5 min after IgE–Ag stimulation, CD63^+^ granules appeared to fuse with each other and form larger intracellular compound granules (Fig. 2a). NLRP3 and ASC strongly colocalized with the CD63^+^ compound granules (Fig. 2a). At 10 min after IgE–Ag stimulation, a time point when the granules were exteriorized, CD63 migrated to the cell periphery along with NLRP3 and ASC (Fig. 2a). Autofluorescence of *Nlrp3*^−/−^ and *Asc*^−/−^ BMMCs was minimal, based on limited staining with ASC-specific and NLRP3-specific antibodies (Extended Data Fig. 2a). Immunoprecipitation assays of wild-type BMMCs indicated a limited association of NLRP3 or ASC with CD63 at steady state, which markedly increased at 3–5 min after IgE–Ag stimulation (Fig. 2b). No association between NLRP3 or ASC with caspase-1 was observed at 3–5 min (Fig. 2b). The post-IgE–Ag stimulation complex comprising NLRP3, ASC and CD63 was distinct from NLRP3-induced canonical inflammasome (ASC and caspase-1; Extended Data Fig. 2b). Moreover, ASC and NLRP3 did not appear to directly bind each other in the IgE–Ag-induced inflammasome complex, which hereafter we refer to as ‘granulosome’, in contrast to the canonical interaction observed after LPS/nigericin treatment of MCs (Extended Data Fig. 2c).

Because the canonical inflammasome is a supramolecular structure of high molecular weight^1^, we performed size exclusion chromatography (SEC) of IgE–Ag-stimulated or LPS/nigericin-treated MC lysates to determine ifthe MC granulosome comprised similar molecular-weight structures. Although the range in size of granulosome fractions suggested they formed high-molecular-weight complexes (400 kDa), they were markedly smaller than inflammasome complexes (600 kDa; Fig. 2c). Caspase-1 was detected in LPS/nigericin-induced inflammasomes, but not in granulosomes (Fig. 2c), while CD63 was the major component of the granulosome but showed minimal association with LPS/nigericin-induced inflammasome complexes (Fig. 2c). Granulosome and inflammasome electrophoretic mobility in IgE–Ag or LPS/nigericin-stimulated MCs indicated that NLRP3 and ASC were mostly found as monomers or dimers in granulosomes, whereas they formed multimeric complexes in inflammasomes (Fig. 2d).

To map the specific NLRP3 and ASC domains that interacted with CD63 to form the granulosome, we compared the ability of Flag-tagged truncated proteins containing the pyrin domain (PYD), nucleotide-binding domain (NBD) or leucine-rich repeat (LRR) domains of NLRP3 or the PYD-CARD-tagged domains of ASC to bind overexpressed CD63 when transfected into HEK293T cells. The NBD domain of NLRP3 and the CARD domain of ASC bound CD63 in this system (Fig. 2e). The CARD domain of ASC binds caspase-1 (ref. 24). Overexpression of CD63 inhibited the association of ASC with caspase-1 in a dose-dependent manner in HEK293T cells (Extended Data Fig. 2d), confirming the CARD domain of ASC bound CD63.

To identify the specific regions on CD63 that bound ASC and NLRP3, we transiently expressed various truncated forms of CD63 in HEK293T cells (Extended Data Fig. 2e). Immunoprecipitation assays indicated that both NLRP3 and ASC specifically bound CD63^TM3+IL^, which contained the transmembrane 3 and internal loop region of CD63, but not other regions, including CD63^TM3^ (TM3 alone; Extended Data Fig. 2e). To examine whether ASC and NLRP3 competed with each other for CD63 binding, we transiently coexpressed CD63^TM3+IL^ with varying doses of NLRP3 or ASC in HEK293T cells. Competitive immunoprecipitation assays indicated that the binding of ASC to CD63^TM3+IL^ could be inhibited by increasing amounts of NLRP3 (Extended Data Fig. 2f), while the binding of NLRP3 to CD63^TM3+IL^ could be inhibited by increasing amounts of ASC (Extended Data Fig. 2f). Because multiple CD63 proteins bind the MC granules, these observations suggested that ASC and NLRP3 bound to different CD63 molecules on the surface of MC granules. To understand how the granulosome complex was stabilized, we examined the behavior of CD63 in IgE–Ag-activated MCs of wild-type, *Nlrp3*^−/−^ and *Asc*^−/−^ BMMCs. CD63 molecules formed both dimers and high-molecular-weight oligomers in wild-type BMMCs (Fig. 2f), but only formed dimers in IgE–Ag-activated *Nlrp3*^−/−^ and *Asc*^−/−^ BMMCs (Fig. 2f), indicating that NLRP3 and ASC molecules bound to dimerized CD63 to promote the formation of stable complexes containing higher oligomers of CD63 in IgE–Ag-activated MCs. Thus, ASC and NLRP3 aggregate on MC granule membranes during MC degranulation, where they initiate CD63 polymerization and the formation of the granulosome.

### NEK7 and Pyk2 kinases initiate granulosome formation

**Extended Data Fig. 3 |:**
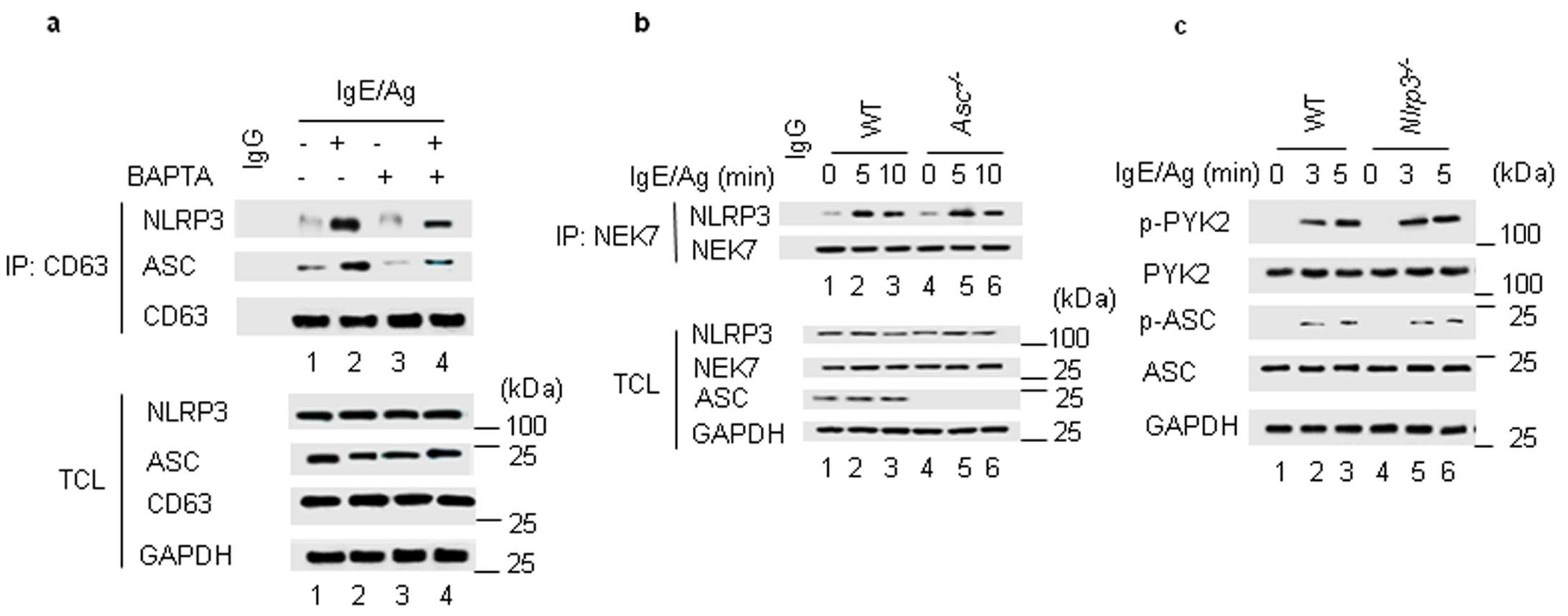
Signaling events downstream of Ca^2+^ axis is important for protein complex formation. **a**, Western blots of immunoprecipitated CD63 with NLRP3, ASC, and tubulin from the lysates, sensitized with IgE and pretreated with or without BAPTA-AM, a chelator of intracellular calcium, following Ag stimulation in WT BMMCs. **b**, Western blots showing endogenous immunoprecipitation of NEK7 from WT and *Asc*^−/−^ BMMCs in response to IgE-Ag stimulation as indicated. The proteins of interests in the total cell lysate (TCL) was also depicted to indicate that similar level of NLRP3, NEK7, and ASC protein present in each fraction. **c**, Western blots showing the status of Pyk2 and ASC activation in IgE-Ag-stimulated WT and *Nlrp3*^−/−^ BMMCs. **c**, ASC is located downstream of Pyk2. Western blots showing the status of Pyk2 and ASC activation in IgE-Ag-stimulated WT and *Nlrp3*^−/−^ BMMCs. Data presented in **a** to **c** are representative of three or more independent experiments.

**Fig. 3 |:**
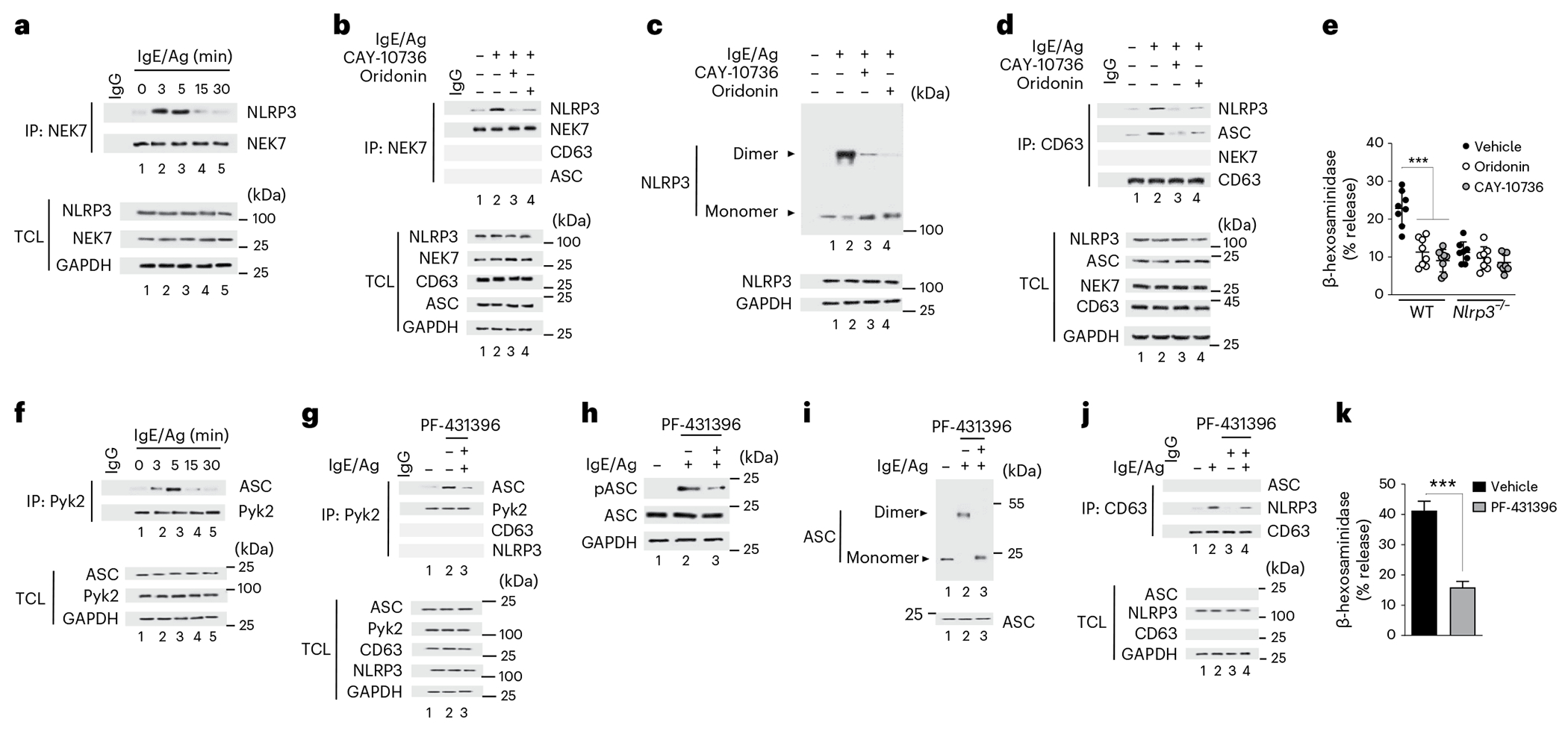
NEK7 and Pyk2 kinases are critical initiators of granulosome formation. **a**, Immunoblotting of NEK7 with NLRP3 from WT BMMCs stimulated with IgE–Ag or left untreated. Protein of interests in the TCL are depicted in each fraction. **b**, Immunoblot of NEK7 with NLRP3, ASC and CD63 in non-stimulated or IgE–Ag-stimulated WT BMMCs pretreated or not with CAY-10736 or oridonin. Protein of interests in the TCL are depicted in each fraction. **c**, Immunoblot analysis of cross-linked NLRP3 from IgE–Ag-stimulated or non-stimulated WT BMMCs pretreated or not with CAY-10736 or oridonin. **d**, Immunoblot analysis of CD63 interaction with NEK7, NLRP3 and ASC in unstimulated or IgE–Ag-stimulated WT BMMCs pretreated or not with CAY-10736 or oridonin. The proteins of interest in the TCL are depicted in each fraction. **e**, Secretion of β-hexosaminidase in WT and *Nlrp3*^−/−^ BMMCs pretreated with vehicle, CAY-10736 or oridonin and stimulated with IgE–Ag. **f**, Immunoblot analysis of PyK2 interaction with ASC in WT BMMCs at 0, 3, 5, 15 and 30 min after stimulation with IgE–Ag. The proteins of interest in the TCL are depicted in each fraction. **g**, Immunoblot analysis of PyK2 interactions with ASC, CD63 and NLRP3 in unstimulated or IgE–Ag-stimulated WT BMMCs pretreated or not with PF-431396. The proteins of interest in the TCL are depicted in each fraction. **h**, Protein immunoblot analysis of phosphorylated ASC from non-stimulated or IgE–Ag-stimulated WT BMMCs pretreated with PF-431396. **i**, Protein immunoblot analysis of cross-linked ASC from WT BMMCs stimulated with IgE–Ag or left untreated. **j**, Immunoblot analysis of CD63 interactions with ASC and NLRP3 in unstimulated or IgE–Ag-stimulated WT BMMCs pretreated or not with PF-431396. The proteins of interest in TCL are depicted in each fraction. **k**, Secretion of β-hexosaminidase from untreated or IgE–Ag-stimulated WT BMMCs pretreated with vehicle or PF-431396. Data shown in **a**–**d** and **f**–**j** are representative of three or more independent experiments. Data shown in **e** are the mean ± s.d. of three independent experiments, performed in duplicate or triplicate, ***P < 0.005 (unpaired, two-tailed Student’s *t*-test), versus WT cells stimulated with IgE/Ag. Data shown in **k** are the mean of three independent experiments, ***P < 0.005 (unpaired, two-tailed Student’s *t*-test), WT cells stimulated with IgE/Ag versus PF-431396 + IgE/Ag.

We next investigated the regulatory signals involved in the formation of granulosomes. Pretreatment of IgE–Ag-stimulated wild-type MCs with BAPTA-AM, an intracellular Ca^2+^ chelator, inhibited the immunoprecipitation of CD63 with ASC and NLRP3 (Extended Data Fig. 3a), suggesting granulosome formation was inhibited. A critical event in NLRP3 inflammasome activation following Ca^2+^ mobilization is the binding of NEK7 to NLRP3 (ref. 5). We found a strong association between NEK7 and NLRP3 (Fig. 3a), but not other granulosome components (Fig. 3b) at 3 min after IgE–Ag stimulation in wild-type BMMCs. The association of NEK7 with NLRP3 was inhibited by CAY-10736, a NEK7 inhibitor^25^, and oridonin, a specific inhibitor of NLRP3 that blocks its binding to NEK7 (ref. 26; Fig. 3b). Pretreatment of IgE–Ag-activated MCs with either CAY-10736 or oridonin markedly inhibited NLRP3 dimerization (Fig. 3c), blocked granulosome formation (Fig. 3d) and MC degranulation (Fig. 3e). Thus, Ca^2+^-triggered and NEK7-mediated dimerization of NLRP3 was an early regulatory signal that led to granulosome formation and MC degranulation.

Dimerization of ASC, which is achieved by tyrosine phosphorylation of ASC, is required for the NEK7-mediated activation of the NLRP3 inflammasome^27^. Pyk2, a non-receptor tyrosine kinase that phosphorylates ASC^6^, is activated in MCs after Ca^2+^ influx^28^. Pyk2 immunoprecipitated with ASC in MCs within 3 min after IgE–Ag stimulation, albeit for a limited period (Fig. 3f). The Pyk2 kinase inhibitor PF-431396 (ref. 29) blocked Pyk2 binding to ASC, as well as the phosphorylation and dimerization of ASC (Fig. 3g–i). While Pyk2 was not a component of granulosomes (Fig. 3g), blocking its interaction with ASC blocked granulosome formation and downstream MC degranulation (Fig. 3j,k). NEK7 bound NLRP3 in IgE–Ag-stimulated *Asc*^−/−^ BMMCs (Extended Data Fig. 3b), while Pyk2 mediated ASC activation in *Nlrp3*^−/−^ BMMCs (Extended Data Fig. 3c). Thus, after IgE–Ag challenge, the rise of Ca^2+^ in the MC cytosol triggered NEK7-dependent dimerization of NLPR3 and the Pyk2-mediated phosphorylation of ASC, which were independent of each other, but together lead to granulosome formation.

### Granulosome trafficking requires MT polymerization

**Extended Data Fig. 4 |:**
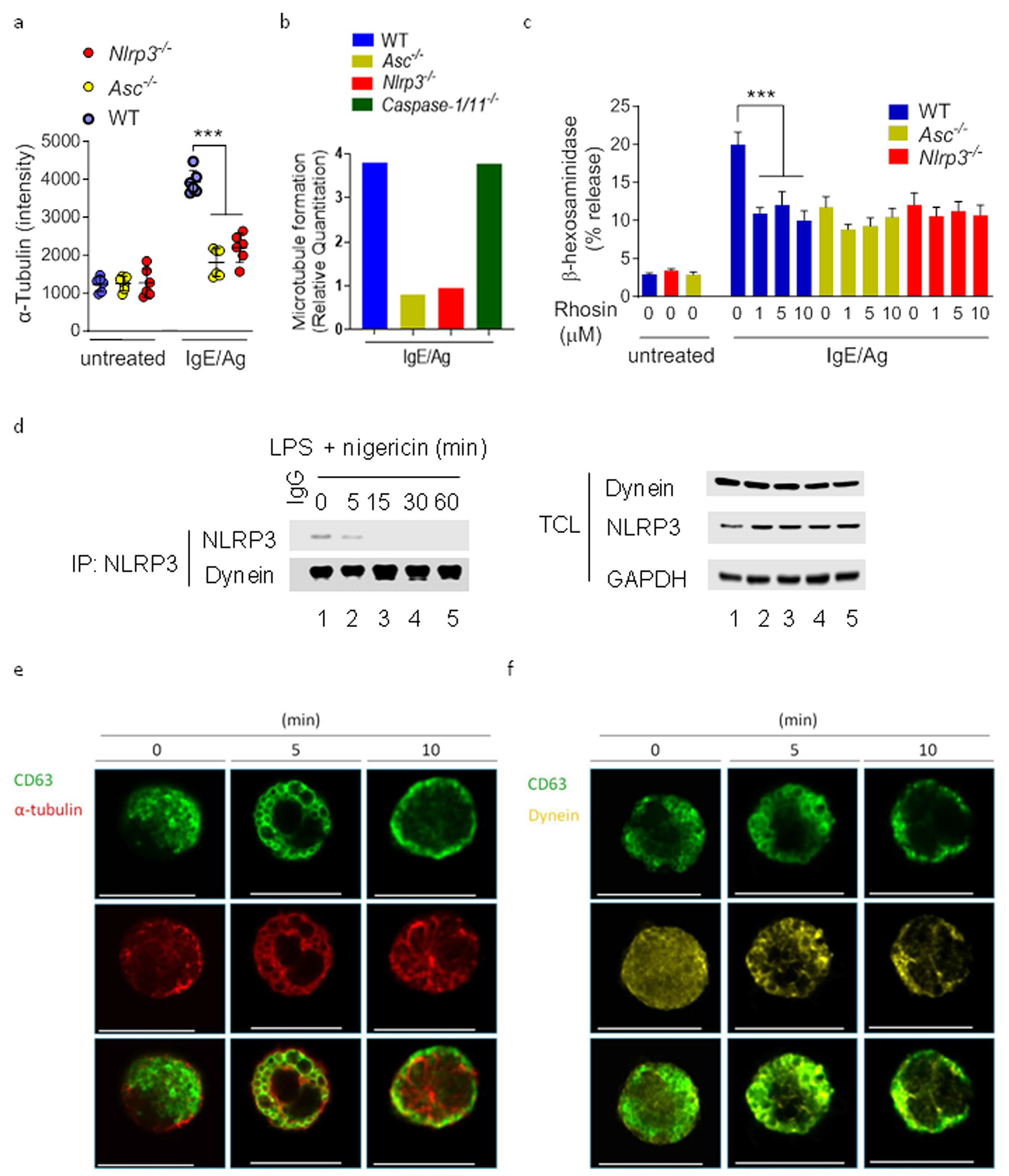
Distribution of tubulin and dynein on MC granules. **a**, Quantitative analysis of α-tubulin in WT, *Nlrp3*^−/−^ and *Asc*^−/−^ BMMCs following IgE-Ag stimulation. The α-tubulin signals were measured in each cell with a radius of 12-16 μm using ZEN Imaging Software (Zeiss). Background fluorescence using a circle of the corresponding size was subtracted from each measurement. **b**, Densitometric analysis of Fig. 4b tubulin protein intensity from WT, *Nlrp3*^−/−^, *Asc*^−/−^, and *Caspase-1/11*^−/−^ BMMCs following IgE-Ag stimulation. **c**, β-hexosaminidase release from untreated or IgE-Ag stimulation in WT, *Nlrp3*^−/−^, and Asc^−/−^, BMMCs pre-treated with varying concentrations of Rhosin, a drug that targets RhoA GTPase. **d**, Western blots showing immunoprecipitation of NLRP3 with dynein from WT BMMCs primed with LPS and subsequently challenged with varying amounts of nigericin. **e**, Representative confocal microscopy images co-stained for CD63 (green) and tubulin (red). **f**, CD63 (green) and dynein (light yellow) in WT BMMCs following IgE-Ag stimulation at different time points. Scale bar: 20 μm. **f**, Densitometry analysis of Fig. 4f protein (GFP-CD63) intensity from siRNAs (control, *Nlrp3*, and *Asc*) of the plasma membrane. Data shown in a are mean ± s.d. of one representative experiments, *n* = 6 cells/group. ***P < 0.001 (Ordinary one-way ANOVA multiple comparisons procedure). Data shown in **b** are densitometry analysis of Fig. 4b and representatives of three independent experiments. Data shown in **c** are mean ± s.d of four independent experiments, performed in triplicate, ***P < 0.005 (Ordinary one-way ANOVA multiple comparisons procedure), vs. WT cells stimulated with IgE/Ag with no Rhosin. Data shown in **d** is representative of two independent experiments. Immunofluorescence images shown in **e** and **f** are representative of two independent experiments with similar results.

**Fig. 4 |:**
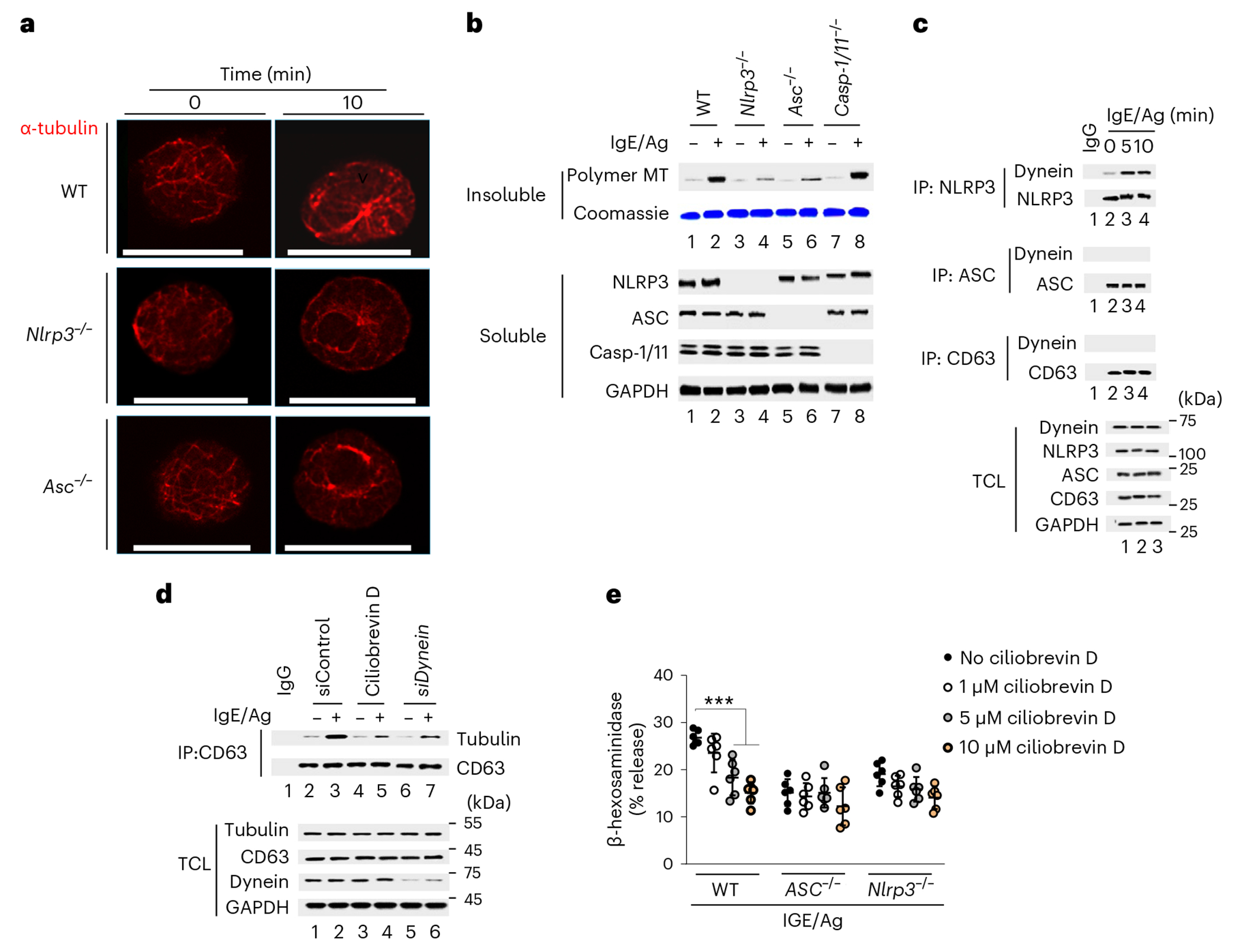
Granulosomes facilitate MT polymerization and granule trafficking. **a**, Immunofluorescence microscopy of α-tubulin staining in WT, *Nlrp3*^−/−^ and *Asc*^−/−^ BMMCs before and 10 min after IgE–Ag stimulation. Scale bars, 20 μm. **b**, Protein immunoblot analysis of insoluble and Triton buffer soluble fractions in WT, *Nlrp3*^−/−^, *Asc*^−/−^ and *Casp-1/11*^−/−^ BMMCs stimulated or not with IgE–Ag with SDS–PAGE gel stained with Coomassie brilliant blue used as an equal loading control. **c**, Immunoblot analysis of NLRP3, ASC or CD63 interactions with dynein in WT BMMCs stimulated with IgE–Ag for 0, 5 or 10 min. The proteins in the TCL are depicted in each fraction. **d**, Immunoblot analysis of CD63 interactions with tubulin in unstimulated or IgE–Ag-stimulated WT BMMCs pretreated or not with ciliobrevin D, control siRNA or dynein siRNA. The proteins of interest in the TCL are depicted in the same fraction. **e**, β-hexosaminidase release in IgE–Ag-stimulated WT, *Asc*^−/−^ and *Nlrp3*^−/−^ BMMCs left untreated or pretreated with 1, 5 or 10 μM ciliobrevin D. Data shown in **b**–**d** are representative of three independent experiments. Data shown in **e** are the mean ± s.d. of two independent experiments, performed in triplicate, ***P < 0.005 (ordinary one-way ANOVA multiple-comparisons procedure), versus WT cells stimulated with IgE/Ag with no ciliobrevin D.

MT reorganization is required for MC degranulation^30^. To investigate the role of granulosomes in MC granule trafficking to the periphery along MTs, we compared the distribution of MTs in IgE–Ag-stimulated wild-type, *Nlrp3*^−/−^ and *Asc*^−/−^ MCs (Fig. 4a and Extended Data Fig. 4a). By 10 min after MC activation, MTs in wild-type BMMCs appeared highly organized, with several strands emanating from the microtubule-organizing center and radiating toward the cell surface (Fig. 4a), presumably to accommodate granule trafficking. Limited organized MT formation was seen in *Nlrp3*^−/−^ and *Asc^−/−^* MCs at this time point (Fig. 4a), with little detectable MTs in the perinuclear region in *Nlrp3*^−/−^ and *Asc*^−/−^ MCs (Fig. 4a). Thus, granulosome formation in wild-type MCs was associated with polymerization of MTs. Immunoblot assays indicated marked increases in the amount of polymeric tubulin associated with granulosomes in wild-type and *Casp-1/Casp-11*^−/−^ BMMCs, but not in *Nlrp3*^−/−^ and *Asc*^−/−^ BMMCs (Fig. 4b and Extended Data Fig. 4b), indicating that granulosomes linked MC granules to MTs.

Rho GTPases are known to promote MT polarization^30–32^. The Rho GTPases inhibitor rhosin A markedly blocked degranulation of IgE–Ag-stimulated wild-type BMMCs but had limited effect in *Nlrp3*^−/−^ and *Asc*^−/−^ BMMCs (Extended Data Fig. 4c). Directional movement on MTs is dependent on motor proteins such as dynein^33^. Immunoprecipitation using NLRP3, ASC or CD63 antibodies indicated NLRP3, but not ASC or CD63, interacted with dynein in IgE–Ag-stimulated wild-type BMMCs (Fig. 4c) but not in nigericin-activated wild-type BMMCs (Extended Data Fig. 4d). Confocal microscopy of wild-type BMMCs revealed the colocalization of endogenous tubulin and dynein around the granule marker CD63 during the early phase (5 min) of MC degranulation (Extended Data Fig. 4e,f) as well as the formation of tubulin filaments and dynein relocation near the periphery at the 10 min time point (Extended Data Fig. 4e,f). Targeted dynein disruption with the dynein inhibitor ciliobrevin D or dynein silencing (siRNA) in RBL-2H3 after IgE–Ag stimulation markedly decreased the IgE–Ag-triggered association of tubulin with CD63 (Fig. 4d). Pretreatment with ciliobrevin D blocked wild-type BMMC degranulation but was mostly ineffective in *Nlrp3*^−/−^ and *Asc*^−/−^ BMMCs after IgE–Ag stimulation (Fig. 4e). Thus, granulosome migration to the cell periphery required dynein-mediated trafficking on polymerized MTs.

### NLRP3-targeting drug protects against anaphylaxis

**Fig. 5 |:**
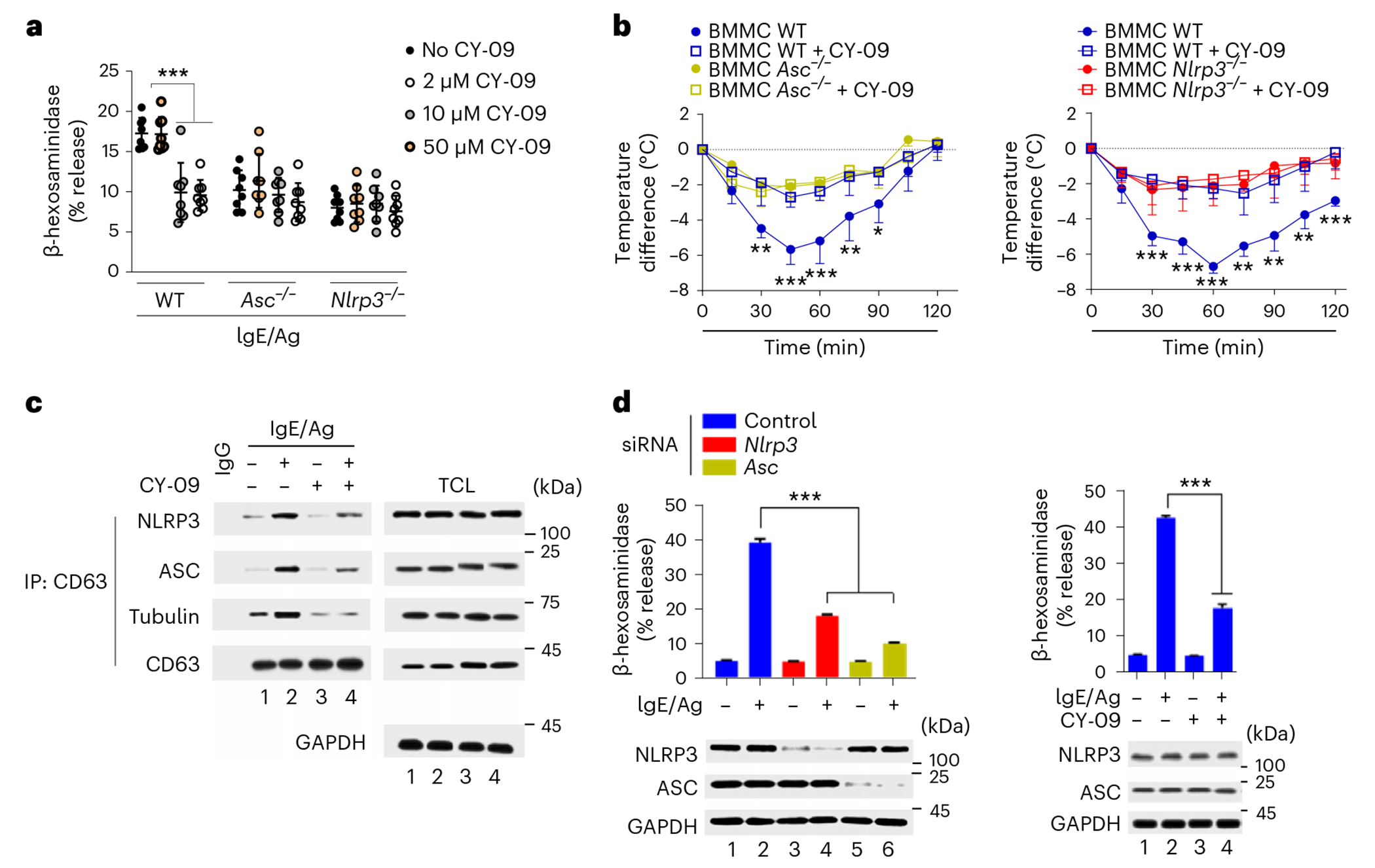
NLRP3-targeting drug protects against anaphylaxis. a, β-hexosaminidase release in WT, *Nlrp3*^−/−^ and *Asc*^−/−^ BMMCs pretreated with 0, 2,10 and 50 μM of CY-09 and stimulated with IgE–Ag (TNP-OVA). **b**, Body temperature changes at 0, 30, 60, 90 and 120 min after Ag administration in IgE-sensitized *Kit_W-sh/W-sh_* mice repleted with WT, *Asc*^−/−^ or *Nlrp3*^−/−^ BMMCs pretreated or not with CY-09 for 1 h. **c**, Immunoblot analysis of CD63 interactions with NLRP3, ASC and tubulin in non-stimulated or IgE–Ag-stimulated WT BMMCs pretreated or not with CY-09. The proteins of interest in TCL are depicted in each fraction. **d**, β-hexosaminidase release from *Nlrp3* KD or *Asc* KD human LAD2 MCs pretreated or not with CY-09 and stimulated or not with IgE–Ag and immunoblots showing NLRP3 and ASC protein expression in TCL. Data shown in a are the mean ± s.d. of three independent experiments, performed in duplicate or triplicate, ***P < 0.005 (ordinary one-way ANOVA multiple-comparisons procedure), versus WT cells stimulated with IgE/Ag with no CY-09. Data shown in **b** are the mean ± s.d. of *n* = 5–6 mice per group, *P < 0.05, **P < 0.01, ***P < 0.005 (unpaired, two-tailed Student’s *t*-test), WT versus WT + CY-09 mice group for each time point indicated. Data shown in **c** are representative of three independent experiments. **d**, Data are the mean ± s.e.m. of one representative experiment, of three independent experiments with similar results, performed in triplicate; ***P < 0.005 in left, siControl + IgE/Ag versus si*Nlrp3* or si*Asc* + IgE/Ag; right, control + IgE–Ag versus CY-09 + IgE/Ag of three independent experiments.

Next, we tested whether drug targeting of NLRP3 abrogated the IgE–Ag-mediated MC degranulation and protected against anaphylaxis. Pretreatment with CY-09, a small-molecule inhibitor shown to be therapeutically efficacious in mouse models of cryopyrin-associated periodic syndrome and type 2 diabetes^34^, specifically blocked the IgE–Ag-mediated degranulation of wild-type, but not *Nlrp3*^−/−^ or *Asc*^−/−^, BMMCs (Fig. 5a). The anaphylaxis response assessed by sharp drop in core body temperature in *Kit^W-sh/W-sh^* mice transferred with wild-type BMMCs and challenged with IgE–Ag was markedly inhibited by CY-09 pretreatment compared to untreated anaphylactic mice, and was similar in magnitude to that observed in CY-09-treated or untreated *Kit^W-sh/W-sh^* mice transferred with *Asc*^−/−^ or *Nlrp3*^−/−^ BMMCs (Fig. 5b), suggesting CY-09 could prevent IgE-mediated anaphylaxis. Because the putative binding site of CY-09 is the NBD of NLRP3, which also bound CD63, we examined whether CY-09 inhibited the binding of CD63 to NLRP3. The association of NLRP3 to CD63 and the binding of ASC and tubulin to CD63 were inhibited in IgE–Ag-stimulated wild-type BMMCs pretreated with CY-09 compared to untreated controls (Fig. 5c).

We also examined whether NLRP3 and ASC were important for the degranulation of human MCs. IgE–Ag stimulation of human LAD2 MC lines in which *Nlrp3* or *Asc* were silenced using siRNA resulted in markedly reduced degranulation compared to MCs transfected with control siRNA (Fig. 5d). Pretreatment for 15 min with CY-09 before IgE–Ag stimulation significantly reduced degranulation in wild-type LAD2 MCs compared to untreated wild-type LAD2 MCs (Fig. 5d), at levels comparable to the degranulation response of IgE–Ag-stimulated, *Nlrp3*-silenced LAD2 MCs (Fig. 5d), suggesting NLRP3 and ASC had similar functions in mouse and human MCs. Thus, CY-09 was a powerful inhibitor of IgE–Ag-mediated MC degranulation in vitro and in vivo.

### Pro-IL-1β is exteriorized via MC granules

**Fig. 6 |:**
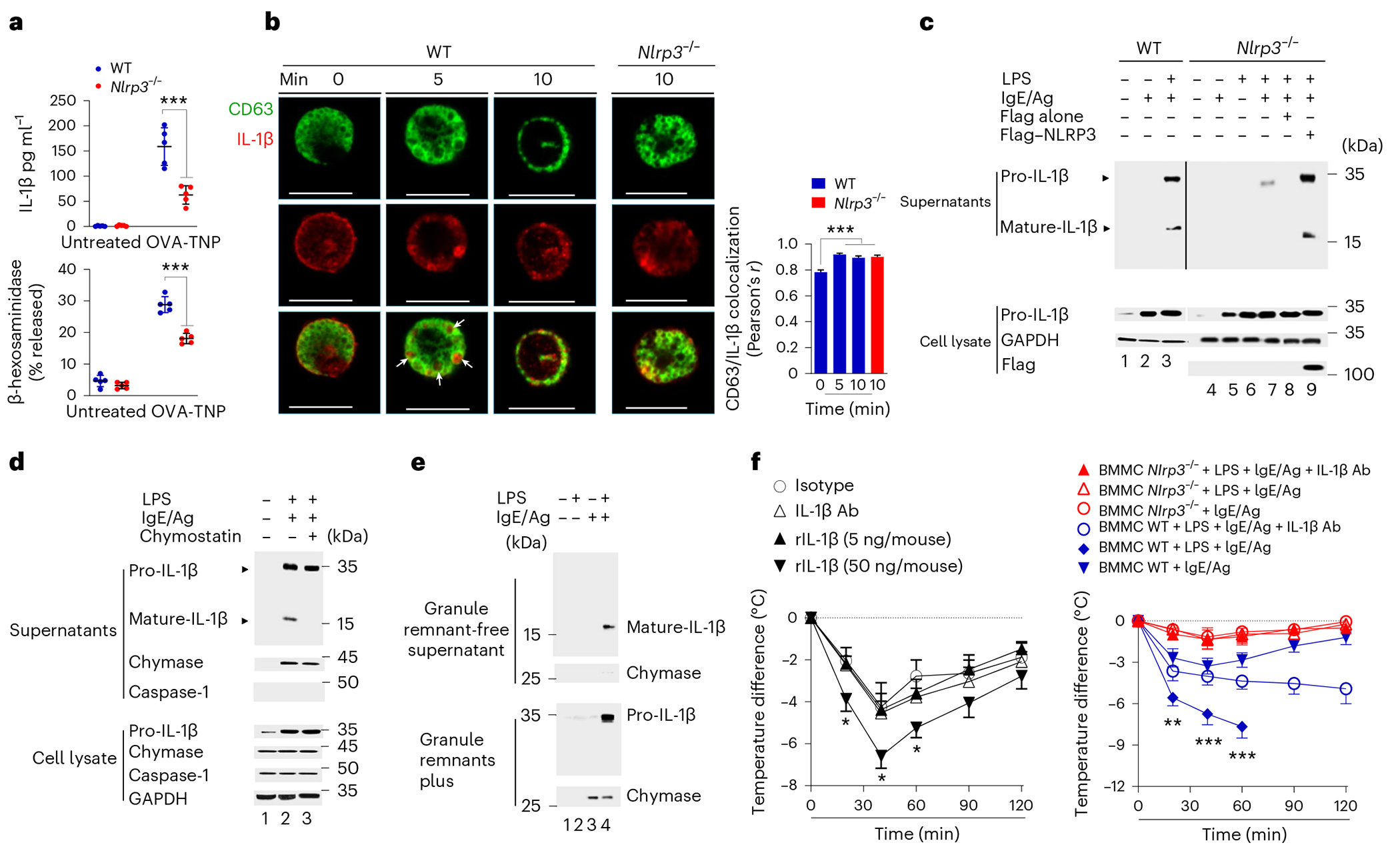
Pro-IL-1β is released from intact granules following IgE–Ag activation of LPS-primed MCs. **a**, ELISA showing release of IL-1β or β-hexosaminidase from WT or *Nlrp3*^−/−^ BMMCs pretreated or not with TNP-specific IgE and LPS followed by stimulation with OVA-TNP (Ag) or not. **b**, Immunofluorescence staining of IL-1β (red) and CD63 (green) in WT or *Nlrp3*^−/−^ BMMCs primed with LPS and TNP-specific IgE for 3–4 h and stimulated with OVA-TNP (Ag) at 0, 5 and 10 min (WT) or 10 min (*Nlrp3*^−/−^) and colocalization of IL-1β with CD63 calculated from 13 representative images using ImageJ (Pearson correlation coefficient). Values are the mean ± s.e.m.; significant differences of treatment versus control were analyzed by one-way ANOVA/Bonferroni’s post hoc test, ***P < 0.001, *n* = 13 images per group. Scale bars, 20 μm. **c**, Immunoblot analysis of pro-IL-1β and mature IL-1β in concentrated supernatants of WT or *Nlrp3*^−/−^ BMMCs activated with IgE–Ag alone or LPS + IgE–Ag and *Nlrp3*^−/−^ BMMCs transfected with Flag or Flag-tagged mouse NLRP3. **d**, Immunoblot analysis of pro-IL-1β and mature IL-1β in the supernatant of non-primed or LPS + IgE-primed Ag-activated WT BMMCs pretreated or not with chymostatin. **e**, Immunoblot analysis of pro-IL-1β and mature IL-1β in the supernatant and granule remnant fractions of WT BMMCs primed or not with LPS and stimulated or not with Ag. **f**, Body temperature changes at 0, 30, 60, 90 and 120 min after Ag administration in WT mice presensitized with TNP-specific IgE antibody (5 μg per mouse) for 2–4 days before Ag challenge (150 μg per mouse) alone or in combination with IL-1β antibody (Ab; 150 μg per mouse) or rIL-1β (5 and 50 ng per mouse; left) and *Kit^W-sh/W-sh^* mice repleted with WT or *Nlrp3*^−/−^ BMMCs sensitized with TNP-specific IgE before LPS pretreatment (i.p. 0.3 μg per mouse) for 90 min followed by i.p. OVA-TNP (Ag) challenge alone or in combination with IL-1β-specific antibody (150 μg per mouse). The data are shown in **a** as the mean ± s.d. of two independent experiments, performed in duplicate or triplicate, ***P < 0.005 by unpaired, two-tailed Student’s *t*-test, as indicated. Immunofluorescence images shown in **b**, and data on the right are representative of three independent experiments, with similar results. The extent of colocalization of IL-1β with CD63 according to images in **b** was calculated from *n* = 13 representative of two independent experiments images using ImageJ (Pearson correlation coefficient). Values are given as the mean ± s.e.m.; significant differences of treatment versus nonactivated IgE–Ag WT BMMCs were analyzed by one-way ANOVA/Bonferroni’s post hoc test, ***P < 0.001, *n* = 13 images per group. The data shown in **c**–**e** are representative of three independent experiments. The data shown in **f** are from *n* = 5–6 mice per group. Left, *P < 0.05; IgE/Ag + isotype versus IgE/Ag + rIL-1β mice group, two-tailed Student’s *t*-test. Right, **P < 0.01, ***P < 0.005; MC-deficient + BMMC WT + IgE + LPS-pretreated groups, Ag challenge alone or in combination with α-IL-1β.

Because NLRP3 and ASC process and release IL-1β, we investigated if they also contributed to IL-1β export in MCs during degranulation. LPS-primed BMMCs stimulated with IgE–Ag^35^, but not IgE–Ag-activated BMMCs, released significant amounts of IL-1β during degranulation (Fig. 6a). IL-1β release was significantly reduced in *Nlrp3*^−/−^ BMMCs, which had markedly diminished degranulation compared to wild-type BMMCs (Fig. 6a). To assess the release of IL-1β in degranulating MCs, wild-type BMMCs that had been primed with LPS for 3 h and then activated with IgE–Ag were immunostained for the presence of IL-1β. Confocal immunofluorescence images were prepared at 0, 5 and 10 min after activation. Whereas IL-1β was mostly found in the cytosol and periphery of BMMCs at time 0, the cytokine appeared packaged within CD63^+^ compound granules 5 min after IgE–Ag stimulation (Fig. 6b). By 10 min after IgE–Ag activation, most of the IL-1β^+^CD63^+^ compound granules had moved to the cell periphery (Fig. 6b), indicating that MCs could rapidly release bulk amounts of IL-1β. IgE–Ag stimulation in LPS-primed *Nlrp3*^−/−^ BMMCs resulted in localization of IL-1β within CD63^+^ compound granules, but the granules did not move to the cell periphery (Fig. 6b). Immunoblotting in cell-free culture supernatant collected from LPS-primed IgE–Ag-stimulated wild-type BMMCs detected high amounts of mature IL-1β and unprocessed pro-IL-1β (Fig. 6c), indicating the majority of IL-1β was unprocessed. LPS-primed *Nlrp3*^−/−^ BMMCs released a minimal amount of pro-IL-1β (Fig. 6c). Transient expression of NLRP3 in LPS-primed *Nlrp3*^−/−^ BMMCs resulted in detection of both immature and mature IL-1β in the culture supernatant (Fig. 6c), indicating that release of processed and unprocessed IL-1β in LPS-primed MCs was dependent on NLRP3.

To address the presence of both pro-IL-1β and mature IL-1β in the culture medium after IgE–Ag-mediated activation of LPS-primed MCs, we investigated whether pro-IL-1β was extracellularly processed by proteases such as chymase, which are borne in MC granules. Immunoblotting detected the presence of chymase, but not caspase-1, in the supernatant of IgE–Ag-stimulated, but not non-stimulated, MCs (Fig. 6d). Pro-IL-1β, but not mature IL-1β, was detected in MC medium treated with chymostatin, a chymase inhibitor (Fig. 6d), indicating that IL-1β processing occurred extracellularly upon MC degranulation. To test whether extracellular processing of pro-IL-1β occurred within intact extracellular granules, as these structures are relatively stable even in the extracellular medium^36^, we separated intact granules from the medium of LPS-primed, IgE–Ag-activated MCs by centrifugation, and compared the amount of pro-IL-1β, mature IL-1β and chymase in pellet and supernatant fractions. Pro-IL-1β and chymase were detected in the intact granule (pellet) fraction, while IL-1β was exclusively detected in the supernatant (Fig. 6e), suggesting that extracellular granules represented the site of pro-IL-1β extracellular processing, before the release of mature IL-1β into the medium.

**Extended Data Fig. 5 |:**
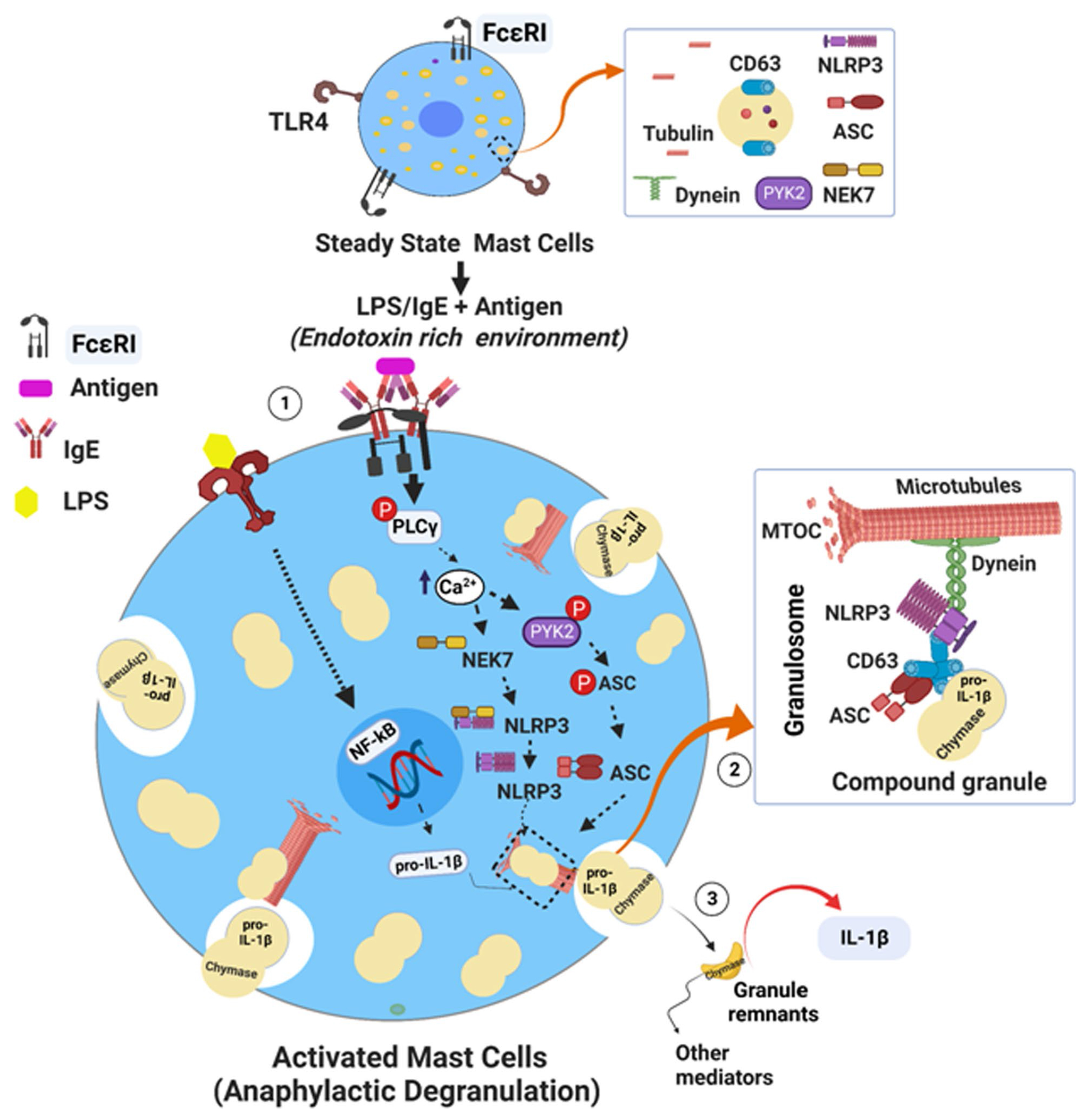
Schematic model showing a cascade of signaling events involved in the formation of granulosome complex during anaphylactic degranulation in MCs. (1) Aggregation of FcεRI by antigen triggers a cascade of mast cell signaling events including the activation of phospholipase C (PLCγ) which in turn increases the intracellular Ca^2+^ levels facilitating the recruitment of two critical kinases, NEK7 and Pyk2. NEK7 kinase binds to NLRP3 and promotes its dimerization, whereas Pyk2 kinase mediates ASC phosphorylation and its dimerization. In many cases, following IgE-Ag activation, granules fuse with each other to form larger compound granules within the mast cell. Thereafter, dimerized NLRP3 and ASC deposit on compound granules enabling a conformational change that allows the oligomerization of CD63 to facilitate the formation of a multiprotein complex ‘granulosome’ comprising NLRP3, ASC, and CD63 (boxed figure) (2). Subsequent to the formation of a microtubule-organizing center (MTOC), a motor protein, dynein is recruited to the granulosome by specifically binding to NLRP3, which then moves along with MTs (microtubules) to the cell periphery where cytosolic compound granules are exteriorized (2). If the MCs are pre-exposed to endotoxin such as LPS, which induces NF-κB-mediated expression of pro-IL-1β(1), thereafter, following IgE-Ag stimulation, pro-IL-1β is rapidly incorporated into the compound granules and when granules are exteriorized, they contain bulk amounts of pro-IL-1β and chymase. In the external medium, pro-IL-1β is processed into mature IL-1β by chymase within the granule remnants (3) before both IL-1β and chymase are released into the external medium. Created with BioRender.com.

To explore the functional consequences of IL-1β release during IgE-mediated MC degranulation in vivo, we compared the magnitude of anaphylaxis assessed by a drop in core body temperature 15 min after challenge with Ag in IgE-sensitized wild-type mice pretreated intraperitoneally (i.p.) with IL-1β-specific antibodies or isotype control. Neither IL-1β antibodies nor isotype treatment significantly impacted the anaphylactic response (Fig. 6f), suggesting MCs did not release IL-1β without LPS treatment. Treatment of IgE-sensitized, Ag-challenged wild-type mice with 50 ng per mouse of recombinant (r)IL-1β significantly enhanced the magnitude of anaphylaxis compared to IgE sensitization and Ag challenge alone (Fig. 6f), while treatment with 5 ng rIL-1β per mouse had no obvious effect (Fig. 6f), indicating that when present in large amounts, IL-1β could markedly potentiate anaphylaxis. Next, MC-deficient *Kit^W-sh/W-sh^* mice transferred with wild-type or *Nlrp3*^−/−^ BMMCs were sensitized with IgE and pretreated with LPS for 90 min before Ag challenge with or without pretreatment with IL-1β-specific antibodies. LPS-treated *Kit^W-sh/W-sh^* mice repleted with wild-type BMMCs experienced anaphylaxis upon Ag challenge and succumbed within 60 min, whereas *Kit^W-sh/W-sh^* mice pretreated with IL-1β-specific antibodies experienced severe anaphylaxis but survived (Fig. 6f). In contrast, LPS-treated *Kit^W-sh/W-sh^* mice repleted with *Nlrp3*^−/−^ BMMCs did not show symptoms of anaphylaxis (Fig. 6f). Thus, IgE–Ag-mediated activation of MCs in the presence of LPS in IgE-sensitized mice triggered a more intense anaphylactic reaction compared to MCs activated in an LPS-free environment, indicating a contribution from the IL-1β packaged in exteriorized granules. Together, these results showed that ASC and NLRP3 contributed to MC degranulation (Extended Data Fig. 5).

## Discussion

We have shown that the inflammasome components NLRP3 and ASC had a crucial and noncanonical role in MC degranulation, by promoting the trafficking of cytosolic granules to the plasma membrane following IgE–Ag-mediated activation. In endotoxin-primed MCs, inflammasome components also facilitated the shuttling and exteriorization of pro-IL-1β, which appeared sequestered within granules immediately after IgE–Ag-induced activation. After degranulation, pro-IL-1β was processed extracellularly within structurally intact granule remnants by MC proteases such as chymase, resulting in the release of mature IL-1β.

When MCs degranulate following activation by IgE–Ag, they rapidly and explosively deliver bulk amounts of prestored bioactive mediators to the surrounding microenvironment. Because MC granules are structurally stable for extended periods following exteriorization^36^, mediators interspersed within granule proteoglycan matrix can be gradually released for sustained periods, prolonging their activity. We found that exteriorized granule remnants could also serve as a matrix for the processing of cytokine precursors such as pro-IL-1β. MC proteases, such as chymase, processed pro-IL-1β into its mature form within granule remnants upon exteriorization. Granule remnants may gradually release various cargo into the extracellular environment, while providing a secure compartment for proteases to process other granule contents without degradation or dilution^37^. The observation that IgE–Ag-triggered and MC-mediated anaphylaxis became lethal in the presence of LPS indicated that MC-derived IL-1β enhanced the potency of IgE-mediated reactions. This observation is consistent with clinical findings that household endotoxin exposure aggravates IgE-mediated asthma and airway allergies^38,39^. The endotoxin-potentiating effect on IgE-mediated MC degranulation responses could have physiologic relevance, as IgE–Ag-activated MCs are critical in expelling helminths from the intestines^40,41^. The endotoxins at this location could provide an additional signal to intensify the inflammatory response against these recalcitrant parasites.

Based on our observations, two seemingly independent enzymatic activities simultaneously occurred in the cytosol following the IgE–Ag-mediated elevation of intracellular Ca^2+^ in MCs. NEK7 bound NLRP3 and Pyk2 phosphorylated ASC to induce their respective dimerization, followed by binding of dimerized NEK7 and Pyk2 to distinct CD63 molecules to induce CD63 polymerization on the granule membrane. The formation of a granulosome complex on the granule membrane surface represented a critical signal that promoted MT polymerization and recruitment of the motor protein dynein. We speculate that NLRP3 and ASC dimers bound different CD63 molecules because they bound the same region on CD63. It is possible that multiple granulosome complexes decorate the membrane of each granule and collaborate to stabilize the coupling of MC granules to MT, as they rapidly traffic to the cell periphery. Although granules in many types of granulocytes are bound by CD63, it is unclear if their degranulation would involve ASC and NLRP3. Neutrophils and basophils, like MCs^19^, constitutively express NLRP3 (ref. 42), suggesting they may also form granulosomes.

Dynein was critical for granule shuttling along MTs, as its loss or inactivation abrogated both the association of CD63 with tubulin and MC degranulation. Compared to other granulosome components, dynein preferentially bound NLRP3, which might be the specific component on the complex that latches onto the motor protein as the compound granules are conveyed along MTs. Although dynein promotes bidirectional transport along MTs^43^, only dynein-mediated retrograde transport of MC granules has been reported so far^33,44^. We found dynein was involved in antegrade transport of MC granules following IgE-mediated activation. While a role for MTs in the intracellular translocation of IL-1β following inflammasome-mediated processing has not been reported, MTs and associated dynein have been implicated in the coupling of NLRP3 to ASC in the early stages of inflammasome formation. Despite the reduced degranulation capacity compared to the wild type, MCs deficient in NLRP3 or ASC still exhibited limited degranulation capability suggesting that not all granules are linked to MTs for exteriorization. Peripherally located granules, because of their proximity to the plasma membrane, are likely exteriorized without the need for NLRP3-ASC-containing complexes and MTs to mediate intracellular conveyance.

Despite the potentially life-threatening nature of IgE-mediated anaphylaxis, there remain limited avenues for treating this severe allergic reaction. NLRP3-targeting molecules have been reported to be effective in reducing harmful inflammation^45^ but so far not against anaphylaxis. We have found that pretreatment with the NLRP3-targeting small-molecule CY-09 was effective in preventing IgE–Ag-evoked anaphylaxis in mice, but it remains to be determined whether it has any therapeutic benefit after anaphylaxis has been triggered.

MCs are ancient innate immune cells predating the emergence of chordates >500 million years ago. Conceivably, NLRP3 and ASC developed in MCs initially to mediate degranulation responses to external threats as the *NLRP3* gene is present in the common ancestor of chordates. Subsequently, MC degranulation machinery adapted to accommodate IL-1β production by facilitating extracellular processing of the cytokine within granules. As other non-granule-forming immune cells evolved, NLRP3 and ASC may have been developed as alternate mechanisms to process IL-1β, including forming a complex that activates intracellular proteases such as caspase-1. That caspase-1 has functional relatives in jawed fish, but not in amphibians^46^, suggests it emerged more recently, after the teleost–tetrapod split happened ~450 million years ago. These evolved MC traits of rapid and extensive degranulation upon stimulation and of altering granule composition in response to environmental cues, such as endotoxins, allow MCs in different body sites to tailor prompt and appropriate responses to external threats.

## Online content

Any methods, additional references, Nature Portfolio reporting summaries, source data, extended data, supplementary information, acknowledgements, peer review information; details of author contributions and competing interests; and statements of data and code availability are available at https://doi.org/10.1038/s41590-024-01788-y.

## Methods

### Mice

MC-deficient mice (*Wsh/Wsh*; ‘*Sash*’) were purchased from Jackson Laboratories and bred in-house at Duke-NUS Medical School vivarium, Singapore. Germ-free mice on the C57BL/6 background were purchased from Biological Resource Center (BRC), Agency for Science, Technology, and Research (A*STAR, Singapore)/InVivos (Singapore), and maintained in a germ-free facility. *Nlrp3*^−/−^ mice were procured from the University of Lausanne (Switzerland) and *Asc*^−/−^ and *Caspase-1*^−/−^ from Genentech (USA) and were maintained at BRC (A*STAR), under specific pathogen-free conditions. All animal experiments were performed in compliance with the Guide for the Care and Use of Laboratory Animals and were approved by the Institutional Animal Care and Use Committee, SingHealth (protocol no. 2015/SHS/1121), and BRC (A*STAR; protocol no. 161113).

### Cell culture

To generate BMMCs for both in vitro experiments and in vivo reconstitution studies, bone marrow was flushed from mouse femurs and cultured in RPMI medium containing 10% FBS (Gibco BRL), 100 U ml^−1^ penicillin, 0.1 mg ml^−1^ streptomycin, HEPES, sodium pyruvate, the non-essential amino acid (Invitrogen, Life Technologies, Singapore) and murine rIL-3 (3 ng ml^−1^; ImmunoTools, 12340033) and stem cell factor (SCF; 3 ng ml^−1^; ImmunoTools, 12343323). Rat basophilic leukemia-2H3 MCs (RBL-2H3) were cultured in α-MEM (Invitrogen, Life Technologies, Singapore) supplemented with 10% FBS, 100 U ml^−1^ penicillin/100 μg ml^−1^ streptomycin (GIBCO/Thermo Fisher, MT30002CI) in a 5% CO_2_ incubator at 37 °C. HEK293T cells were cultured in α-DMEM (Invitrogen, Life Technologies, Singapore) supplemented with 10% FBS (Invitrogen, Life Technologies, Singapore), 100 U ml^−1^ penicillin and 100 μg ml^−1^ streptomycin in a 5% CO_2_ incubator at 37 °C. LAD2 human MCs were propagated in complete StemPro-34 medium (Thermo Fisher, 10639011) supplemented with 100 ng ml^−1^ recombinant human SCF (R&D Systems, 255-SC-010/CF).

### In vivo mouse studies

Reconstitution of *Sash* mice was performed by injecting 1 × 10^7^ BMMCs i.v. followed by a 12–16-week period before use in experiments to allow full engraftment, as previously described^36^. For passive systemic anaphylaxis studies, mice were i.p. injected with anti-TNP IgE (5 μg per mouse; BD Biosciences, 557080) 2–4 days before the challenge i.v. with TNP-OVA (250 μg per mouse; Biosearch Technologies, T-5051). Body temperature was recorded using an intrarectal probe (Physitemp, BAT-12) at the times indicated. The in vivo CY-09 (Sigma-Aldrich, SML2465) selective inhibitory activity of MCs NLRP3/ASC was confirmed by the passive systemic anaphylaxis using MC-deficient mice repleted with BMMCs from WT, *Asc*^−/−^ or *Nlrp3*^−/−^ mice sensitized with anti-TNP IgE and pretreated or not with CY-09 (100 μg per mouse i.p.) 1 h before the challenge with TNP-OVA. To investigate the contribution of IL-1β in passive systemic anaphylaxis after the anti-TNP IgE sensitization (5 μg per mouse), mice were injected i.p. with TNP-OVA (150 μg per mouse) alone or with murine rIL-1β (5 or 50 ng per mouse; ImmunoTools, 12340012) or purified anti-mouse IL-1β antibody (150 μg per mouse; clone B122, BioLegend, 515801). In another set of experiments, LPS (Sigma-Aldrich, L2880) pretreatment i.p. (0.3 μg per mouse) was done or not, 90 min before the i.p. OVA-TNP challenge in combination with anti-mouse IL-1β antibody (150 μg per mouse). Purified Armenian Hamster IgG antibody (150 mg per mouse; clone HTK888, BioLegend, 400911) was used as an isotype control.

### MC degranulation assay

WT, *Nlrp3*^−/−^, *Asc*^−/−^ BMMCs (2 × 10^5^ per well) were sensitized overnight at 37 °C with a 0.5 μg ml^−1^ anti-DNP IgE antibody in a 96-well plate and were stimulated for 30 min with various concentrations of DNP-BSA (Sigma-Aldrich). Where indicated, the cells were pretreated for 15–30 min with Ciliobrevin D (Merck Millipore, 250401), CY-09 (Sigma-Aldrich, SML2465) or CAY-10736 (Cayman, Cay26754-1) or oridonin (Sigma-Aldrich, O9639) or PF-431396 (Sigma-Aldrich, PZ0185) or rhosin (Sigma-Aldrich, 555460-25MG-M). Samples were centrifuged, and the supernatant was collected to measure the released β-hexosaminidase (Sigma-Aldrich, N9376) and histamine (Enzo Life Sciences, ENZ-KIT140-001). To evaluate the total cell count of β-hexosaminidase, cells were lysed in 0.5% Triton X-100 in PBS buffer. For the degranulation assay, 100 μl of supernatants or cell lysates were incubated with 50 μl p-nitrophenyl-*N*-acetyl-β-d-glucosaminide (1.3 mg ml^−1^ in 0.1 M sodium citrate, pH 4.5), and incubated for 1 h at 37 °C. The reaction was stopped by the addition of 150 μl 0.1 M carbonate buffer, pH 10, and measured at OD_405_ nm with a microplate reader (Tecan). The percentage of degranulation was calculated by dividing absorbance in the supernatant by the sum of absorbance in the supernatant and cell lysate.

### Knockdown of *NLRP3*, *ASC* and *Caspase-1* in RBL-2H3 MCs

RBL-2H3 cells (500,000 cells per well) were transfected with 100 nM of each siRNA procured from Dharmacon containing control (D-001810-01) or *Asc* (L-097663-02) or *Nlrp3* (L-084509-02) or Caspase-1 (AM16708) individually or in combination 100 nM of each siRNA (*Nlrp3* + *Asc*) in 24-well dishes using X-Fect (Clontech, 631317) according to the manufacturer’s instructions (Dharmacon). After 48 h, the cells were stimulated with IgE–Ag (0.5 μg ml^−1^)/Ag (1 μg ml^−1^) for 45 min at 37 °C, and MC degranulation was monitored as described in ‘MC degranulation assay’.

### Ectopic expression of NLRP3 and ASC in *Nlrp3*^−/−^ or *Asc*^−/−^ BMMCs

BMMCs obtained from either *Nlrp3*^−/−^ or *Asc*^−/−^ (2 × 10^7^) or WT were nucleofected with 5 μg of each expression plasmid expressing mouse Flag-tagged NLRP3 (Addgene, 75127) or ASC (Addgene, 75134) or empty plasmid using P3 Primary Cell 4D-Nucleofector X-kit (Lonza). After 36 h, the MCs were stimulated with IgE–Ag (0.5 μg ml^−1^)/Ag (1 μg ml^−1^). Degranulation was measured by the release of β-hexosaminidase levels in the culture medium as described in ‘MC degranulation assay’.

### Calcium measurement assay

The Ca^2+^ influx was determined in BMMCs from WT, *Nlrp3*^−/−^ and *Asc*^−/−^ BMMCs loaded with Fluo-4 NW probe (Thermo Scientific, F36206) following the manufacturer’s instructions. Cells were stimulated with IgE–Ag or thapsigargin (100 nM; Sigma-Aldrich, T9033). Fluorescence intensities were measured using a Tecan spectrophotometer.

### Measurements of MC-derived mediators

BMMCs were seeded after anti-DNP IgE antibody sensitization at a concentration of 3 × 10^5^ per well at a well volume of 200 μl and stimulated with DNP-BSA (0.5 μg ml^−1^), or primed with LPS (250 ng ml^−1^ for 3–4 h) and stimulated with nigericin (Sigma-Aldrich). The cell culture supernatants were harvested and subjected to ELISA quantification for TNF, IL-6, IL-1β (e-Bioscience, BMS607-3, 29-8061-65, BMS6002), histamine (Enzo Life Sciences, ENZ-KIT140-001) and PGD_2_ (Cayman Chemical, 512031). IL-1β was also quantified in the supernatant of WT and *Nlrp3*^−/−^ BMMCs (2 × 10^6^ per well per ml) with or without TNP-OVA (0.5 μg ml^−1^) treatment for 30 min after anti-TNP IgE (0.5 μg ml^−1^) and LPS (250 ng ml^−1^) primed for 4 h.

### Flow cytometry

For CD63 externalization, MCs were fixed (4% paraformaldehyde) for 20 min at room temperature, then rinsed three times in PBS for 5 min and incubated with α-CD45, α-FcεRI, α-CD117 and α-CD63 (BioLegend; 1:100 dilution) for 30 min at 4 °C. For the detection of MC maturation, cells were resuspended in PBS without Ca^2+^ and Mg^2+^ containing 2 mM EDTA, 2% FBS and stained with CD45, FcεRI and CD117 (BioLegend), followed by fixation, permeabilization with BD Cytofix/Cytoperm Fixation/Permeabilization Solution Kit (BD Bioscience) and intracellularly stained for MC granules using the granule heparin-specific probe avidin-FITC conjugated (BD Pharmingen) for 20 min. MC granularity was confirmed to be >95% pure by toluidine blue (Sigma-Aldrich).

### Immunofluorescence microscopy

Cells were fixed in 4% paraformaldehyde for 20 min, then rinsed three times in PBS for 5 min and incubated in blocking buffer (0.1% saponin (Sigma-Aldrich, 47036) in 1% BSA-PBS) for 30 min at room temperature. The cells were then incubated with α-mouse CD63 (MBL, rat IgG; 1:100 dilution; D263-3) or α-mouse ASC (EMD Millipore, mouse IgG; 1:1,000 dilution; 04-147) or α-mouse NLRP3 (Novus Biological, rabbit IgG ; 1:50 dilution; NBP2-12446) or α-mouse α-tubulin (Sigma-Aldrich, mouse IgG; 1:250 dilution; T5168) or α-mouse α-dynein (Sigma-Aldrich, mouse IgG; 1:250 dilution; MAB1618) or α-mouse IL-1β (Abcam, rabbit IgG; 1:100 dilution; ab9722) diluted in blocking buffer for 3 h. Cells were then washed twice in blocking buffer for 5 min and incubated overnight at 4 °C in blocking buffer. The next day, the cells were washed once in blocking buffer for 5 min and incubated with the following secondary antibodies: goat α-rat IgG AF488 (Thermo Scientific, A-11006) for CD63, chicken α-rabbit IgG AF568 (Abcam, ab175470) for NLRP3 and IL-1β, donkey α-mouse IgG AF647 (Abcam, ab6706) for ASC, donkey α-mouse IgG AF568 (Abcam, ab175472) for dynein and chicken anti-mouse IgG AF568 (Abcam, ab175473) for α-tubulin detection, for 1 h at room temperature, diluted at a 1:400 ratio in blocking buffer. Cells were then washed 4–5 times, incubated overnight at 4 °C in blocking buffer, and mounted. All the images were acquired on an LSM 710 Carl Zeiss microscope using a Plan-Apochromat ×63/1.40 oil DIC objective. Images were acquired at 16-bit depth at a resolution of 1,024 × 1,024 pixels. The α-tubulin signals were measured in each cell with a radius of 12–16 μm using ZEN Imaging Software (Zeiss). Background fluorescence using a circle of the corresponding size was subtracted from each measurement.

### Endogenous and ectopic protein interaction assays and immunoblotting

For analyzing endogenous protein interactions, IgE-sensitized WT, *Nlrp3*^−/−^ or *Asc*^−/−^ BMMCs (6 × 10^7^ cells) were pretreated with CAY-10736 (10 μM) or oridonin (10 μM) or BAPTA-AM (10 μM; Sigma-Aldrich, A1076) or PF-431396 (10 μM) for 30 min following Ag, DNP-BSA (1 μg ml^−1^) or LPS/nigericin stimulation at different time intervals. The cells were washed in ice-cold PBS and lysed in 1× IP lysis buffer (Cell Signaling, 9803) containing 1 mM phenylmethylsulfonyl fluoride (Sigma-Aldrich, P7626-1G), 1 mM sodium orthovanadate (Sigma-Aldrich, S6508) and 1× protease/phosphatase inhibitors (Roche). TCL (1 mg protein equivalent) were incubated with G-plus agarose beads (25 μl; Santa Cruz, sc-2002) for 2 h. The pre-cleared cell lysates were immunoprecipitated with 1 μg of each pull-down antibody, CD63 (Santa Cruz, sc-5275), NLRP3 (Novus Biologicals), ASC (Santa Cruz, sc-514414) and/or NEK7 (Novus Biologicals) and Pyk2 (Cell Signaling, 3480), and tumbled overnight at 4 °C. The following day, immunocomplexes were precipitated with 30 μl of fresh G-plus agarose beads. After 6–8 h, immune complex precipitates were then extensively washed three times with ice-cold lysis buffer. These precipitates or TCL were then subjected to 12% SDS–PAGE and immunoblotted with corresponding antibodies. Anti-IgG of either rabbit (Abcam, ab37415) or mouse (Abcam, ab37355) origin was used as an isotype control. The images were captured using a GelDoc touch screen (Bio-Rad).

For analyzing overexpression protein interaction studies, GFP-CD63 (10 μg; Sino Biological, MG50557-ANG), Flag–NLRP3/Flag–PYD/Flag–NBD/Flag–LRR (10 μg each; Addgene, 75137, 75140, 75141), and Flag–ASC/Flag–PYD/Flag–CARD truncate (10 μg each; Addgene, 75134); or GFP-CD63 full length/or its truncates, GFP-tagged TM1, TM2, TM3 and TM4, or 1 and 5 μg of Flag-tagged NLRP3 or Myc-tagged ASC with a constant amount of GFP-CD63-TM3 (5 μg) were transfected into HEK293T cells (700,000 cells per well) using JetPrime transfection reagent (Polyplus). After 36 h, cell lysates were lysed and incubated with 1 μg of Flag (Cell Signaling, 8146) or GFP (Santa Cruz, sc-9996) or Myc (Cell Signaling, 5605) antibodies. The immune complexes were washed thrice in 1× IP Wash buffer, and eluted with 4X SDS-loading dye, and separated onto 4–20% SDS–PAGE (Bio-Rad) followed by immunoblot analysis with the Flag or Myc or GFP antibodies, respectively.

For analyzing total/phosphorylation status of the respective signaling molecules, the following antibodies were procured from Cell Signaling (used in a 1:1,000 dilution); Syk (3198), pSyk (Tyr525/526) 2710), PLCγ (2822), pPLCγ (14008), ERK1/ERK2 (4695), pERK1/ERK2 (4377), p38 (8690), p-p38 (4511), JNK (9252), pJNK (9255), Pyk2 (3292S), pPyk2 (3291), NEK7 (Novus Biological, NBP-31110) or pASC (ECM, AP5631, AP5631). For WT or *Nlrp3*^−/−^ or *Asc*^−/−^ BMMCs pretreatment with or without PF-431396 following IgE–Ag stimulation at different time intervals, the cell lysates were lysed in Western buffer (150 mM NaCl, 20 mM Tris-HCl, pH 7.4, 1 mM EDTA plus freshly prepared protease inhibitors), and incubated on ice for 30 min. The samples were centrifuged at 14,000*g* for 10 min. The lysates were quantified using Bradford’s assay. An equal number of protein lysates were mixed with 2× SDS-loading dye and boiled at 100 °C for 10 min. The samples were fractionated onto 4–20% SDS–PAGE gels (Bio-Rad) and subjected to immunoblot analysis.

### SEC

WT BMMCs (5 × 10^7^ per ml) were stimulated with IgE–Ag for 5 min or LPS (12 h)/nigericin (Sigma-Aldrich) for 15 min. The cells were homogenized with the protein extraction buffer A (20 mM 4-(2-hydroxyethyl)-1-piperazineethanesulfonic acid-KOH (pH 7.5), 10 mM KCl, 1.5 mM MgCl_2_, 1 mM Na-EDTA and 1 mM EGTA), and freshly prepared protease inhibitor cocktail (Roche). Samples were then centrifuged at 18,000*g* for 10 min at 4 °C. Sephacryl 100 HR SEC matrix (Sigma-Aldrich, S100HR) was pre-equilibrated in buffer B (50 mM Tris-HCl (pH 7.4), 150 mM NaCl, 1% octyl glucoside plus protease inhibitor cocktail. Protein standards (IgM, 970 kDa; Thyroglobulin, 669 kDa; β-amylase, 200 kDa; BSA, 66 kDa, cytochrome C, 12 kDa) were procured from Sigma-Aldrich (MWGF1000) and were run on a column, and the fractions were monitored at A_260_ nm. The column was washed and the clear supernatant (100 μl) obtained from control or IgE–Ag or LPS/nigericin (Sigma-Aldrich, N7143) treatments was loaded onto the SEC column separately. Fractions (100 μl) were collected, starting at the void volume time. An equimolar amount (45 μl) of the fractions were mixed with 4× SDS-loading dye, boiled at 100 °C for 10 min, and then fractionated onto 4–20% SDS–PAGE gels followed by immunoblot analysis with NLRP3, ASC, Caspase-1 and CD63 antibodies.

### Oligomerization assay

WT BMMCs (1 × 10^7^ per ml) were either sensitized with IgE (0.5 μg ml^−1^) or primed with LPS (50 ng ml^−1^) for 12 h. The medium was then replaced, and cells were stimulated with nigericin (10 μM) or Ag (OVA-DNP, 1 μg ml^−1^) for 15 or 5 min, respectively. The supernatants were removed; cells were rinsed in ice-cold PBS and then lysed by NP-40 for 30 min. Lysates were centrifuged at 330*g* for 10 min at 4 °C. The pellets were washed gently twice in 1 ml of ice-cold 1× PBS and resuspended in 500 μl of PBS. Disuccinimidyl suberate (2 mM; Sigma-Aldrich, 225827) was added to the resuspended pellets and incubated at room temperature for 30 min with gentle rotation. Samples were then centrifuged at 330*g* for 10 min at 4 °C. The cross-linked pellets were resuspended in 30 μl sample buffer, boiled and analyzed by immunoblot using antibodies against NLRP3, ASC, and CD63.

### MT polymerization assay

BMMCs (4 × 10^7^) obtained from WT, *Nlrp3*^−/−^, *Asc*^−/−^ and *Caspase-1/Caspase-11*^−/−^ mice were stimulated with IgE–Ag for 5 min and subjected to polymerization assay as described previously. Briefly, cells were lysed in buffer A (250 mM sucrose, 10 mM HEPES (pH 7.8), 10 mM KCl, 2 mM MgCl_2_, 0.1 mM EGTA, 1 mM dithiothreitol and 0.1% NP-40) and freshly prepared protease inhibitors. The lysates were centrifuged for 10 min at 700*g*, and the supernatants were centrifuged for 10 min at 12,500*g*. The resultant supernatants were used as the cytosolic fraction (soluble), and the pellets were resuspended in 10 mM HEPES buffer (pH 7.4) containing 0.5% NP-40, 50 mM KCl, 150 mM NaCl, 1.5 mM MgCl_2_, 1 mM EGTA and protease inhibitors. Then, the resuspended pellets were recentrifuged, and the supernatants were used for the membrane-enriched fraction (insoluble). Subcellular fractionated lysates were lysed in 4× SDS-loading dye, boiled and briefly centrifuged. An equal number of samples were loaded onto 4–20% (Bio-Rad) SDS–PAGE gels, and analyzed by immunoblot. Insoluble samples were separately stained with Coomassie brilliant blue and served as a loading control.

### Inhibition of dynein in RBL-2H3 MCs

RBL-2H3 cells were silenced for dynein (200 nM) or control siRNA (200 nM) using an X-Fect transfection reagent. Four different siRNA duplexes were designed against different regions of the DHC sequence using Dharmacon’s custom SMARTpool siRNA service (rat cytoplasmic DHC1, 5′-GGGAGGAGGTTATGTTTAA, 5’-GGTGACAGCTTTCGAATGA, 5′-CCAAATACCTACATTACTT, 5′-GCTCAAACATGACAGAATT). The non-targeted duplex III (Dharmacon, D-001810-0X) was used as control. After 36 h, cells were primed with IgE (0.5 μg ml^−1^) overnight, and subsequently pretreated with or without ciliobrevin D (10 μM), for 30 min, and then stimulated with OVA-DNP (1 μg ml^−1^) Ag for 5 min. The lysates were immunoprecipitated with the CD63 antibody and subjected to immunoblot analysis using Tubulin, CD63 and GAPDH (Cell Signaling, 9482) antibodies. TCL were loaded as a control IP input and the silencing of dynein (Santa Cruz, sc-13524).

### Knockdown of *NLRP3* and *ASC* in human LAD2 MCs

LAD2 MCs were maintained in complete StemPro-34 medium supplemented with 100 ng ml^−1^ recombinant human SCF (R&D Systems, 255-SC-010/CF). LAD2 cells (5 × 10^7^) were nucleofected with 200 nM of each siRNA targeting *Nlrp3*, *Asc* or scrambled/control using P3 Primary Cell 4D-Nucleofector X-kit (Lonza, V4XP-3032). siRNAs were custom synthesized from IDT Technologies (Singapore). After 36 h, the LAD2 cells were sensitized with biotinylated human IgE (100 ng ml^−1^; BioLegend, 325503) overnight, and the cells were washed with HEPES (10 mM HEPES, pH 7.4, 137 mM NaCl, 2.7 mM KCl, 0.4 mM Na_2_HPO_4_, 5.6 mM glucose, 1.8 mM CaCl_2_ and 1.3 mM MgSO_4_) containing 0.02% BSA, and/or pretreated with CY-09 (10 μM) for 15 min following Ag (100 ng ml^−1^; Streptavidin, S4762) stimulation for 30 min at 37 °C. Degranulation was measured by the release of β-hexosaminidase levels in the culture medium as described under degranulation assay.

### Phenol–chloroform precipitation of cell-free culture supernatants

For detecting IL-1β (pro- or mature) forms in the extracellular medium, cell culture supernatants (500–1,000 μl) from WT or *Nlrp3*^−/−^ BMMCs from various stimulations were subjected to phenol:chloroform precipitations. Briefly, the supernatant was precipitated by adding a quarter volume of chloroform and 1 volume of methanol. The mixture was centrifuged for 5 min at 12,000 rpm. The top layer containing methanol was aspirated out and replaced with a fresh 1 volume of methanol. The mixture was again centrifuged for 5 min at 12,000 rpm. The pellet obtained was air-dried (10–15 min) and dissolved in 1× Western buffer. Alternatively, WT BMMCs cells stimulated with LPS + IgE–Ag were centrifuged at 200*g* for 2 min. The supernatant obtained was further centrifuged at 14,000*g* for 10 min. The pellet fraction obtained is the granule remnant plus fraction, and the supernatant is the granule remnant-free fraction. The granule remnant plus fraction was further processed using gentle sonication (to break down the membranes of MC granules). These two fractions were subjected to precipitation as described above. The precipitated sample (typically 50 μl) was analyzed by 4–20% SDS–PAGE (Bio-Rad) and immunoblotting using pro-IL-1β (Abcam, ab8722) and Chymase (Abcam, ab233103) antibodies.

### Densitometric analysis

The chemiluminescent blots were imaged first with the ChemiDoc (Bio-Rad). The Band Analysis tool of ImageLab software version 4.1 (Bio-Rad) was used to select and determine the band’s intensity in all blots.

### Statistical analysis

Statistical analyses were performed using Prism version 9 (GraphPad Software). Ordinary one-way ANOVA multiple-comparisons procedure was used for comparisons of more than two groups. A two-tailed, unpaired Student’s *t*-test was used to compare two groups of data. *P* values < 0.05 were considered significant.

### Reporting summary

Further information on research design is available in the Nature Portfolio Reporting Summary linked to this article.

## Extended Data

## Supplementary Material

1

The online version contains supplementary material available at https://doi.org/10.1038/s41590-024-01788-y.

## Data Availability

Source data are available on figshare at https://doi.org/10.6084/m9.figshare.21856569.
