# Supplementary material for: Anaphylactic degranulation by mast cells requires the mobilization of inflammasome components
Source: Nat Immunol. Author manuscript; Available in PMC 2026 Jun 5. (PMC13239758; doi:10.1038/s41590-024-01788-y)
Supplement: 1 [file NIHMS2176282-supplement-1.pdf]

Corresponding author(s): Soman Abraham
Last updated by author(s): 25 February 2024

Reporting Summary

Nature Portfolio wishes to improve the reproducibility of the work that we publish. This form provides structure for consistency and transparency in reporting. For further information on Nature Portfolio policies, see our Editorial Policies and the Editorial Policy Checklist.

Please do not complete any field with "not applicable" or n/a. Refer to the help text for what text to use if an item is not relevant to your study.
For final submission: please carefully check your responses for accuracy; you will not be able to make changes later.

Statistics

For all statistical analyses, confirm that the following items are present in the figure legend, table legend, main text, or Methods section.

n/a	Confirmed	
☐	☑	The exact sample size (*n*) for each experimental group/condition, given as a discrete number and unit of measurement
☐	☑	A statement on whether measurements were taken from distinct samples or whether the same sample was measured repeatedly
☐	☑	The statistical test(s) used AND whether they are one- or two-sided<br>*Only common tests should be described solely by name; describe more complex techniques in the Methods section.*
☑	☐	A description of all covariates tested
☑	☐	A description of any assumptions or corrections, such as tests of normality and adjustment for multiple comparisons
☐	☑	A full description of the statistical parameters including central tendency (e.g. means) or other basic estimates (e.g. regression coefficient) AND variation (e.g. standard deviation) or associated estimates of uncertainty (e.g. confidence intervals)
☐	☑	For null hypothesis testing, the test statistic (e.g. *F*, *t*, *r*) with confidence intervals, effect sizes, degrees of freedom and *P* value noted<br>*Give P values as exact values whenever suitable.*
☐	☑	For Bayesian analysis, information on the choice of priors and Markov chain Monte Carlo settings
☑	☐	For hierarchical and complex designs, identification of the appropriate level for tests and full reporting of outcomes
☐	☑	Estimates of effect sizes (e.g. Cohen's *d*, Pearson's *r*), indicating how they were calculated

Our web collection on statistics for biologists contains articles on many of the points above.

Software and code

Policy information about availability of computer code

Data collection	N/A
Data analysis	ZEN microscopy (Zeiss) and ImageJ software for image analysis; FlowJo v10 for flow cytometry; The Band Analysis tool of ImageLab software version 4.1 (BioRad) for WB.; GraphPad Prism version 9 (GraphPad Software) for statistics.

For manuscripts utilizing custom algorithms or software that are central to the research but not yet described in published literature, software must be made available to editors and reviewers. We strongly encourage code deposition in a community repository (e.g. GitHub). See the Nature Portfolio guidelines for submitting code & software for further information.

Data

Policy information about availability of data

All manuscripts must include a data availability statement. This statement should provide the following information, where applicable:

- Accession codes, unique identifiers, or web links for publicly available datasets
- A description of any restrictions on data availability
- For clinical datasets or third party data, please ensure that the statement adheres to our policy

Mencarelli, Andrea (2024). Raw Data NI-A35223. figshare. Dataset. https://doi.org/10.6084/m9.figshare.21856569

Research involving human participants, their data, or biological material

Policy information about studies with human participants or human data. See also policy information about sex, gender (identity/presentation), and sexual orientation and race, ethnicity and racism.

Reporting on sex and gender	N/A
Reporting on race, ethnicity, or other socially relevant groupings	N/A
Population characteristics	N/A
Recruitment	N/A
Ethics oversight	N/A

Note that full information on the approval of the study protocol must also be provided in the manuscript.

Field-specific reporting

Please select the one below that is the best fit for your research. If you are not sure, read the appropriate sections before making your selection.

☑ Life sciences ☐ Behavioural & social sciences ☐ Ecological, evolutionary & environmental sciences

For a reference copy of the document with all sections, see nature.com/documents/nr-reporting-summary-flat.pdf

Life sciences study design

All studies must disclose on these points even when the disclosure is negative.

Sample size	Sample sizes were chosen based on previous studies. No statistical methods were used to predetermine sample size. All mice were included in the analyses. Mice were randomly allocated to the different groups on the basis of cage, genotype, and litter size. For all experiments, we aimed to have the same number of mice in the control and experimental groups.
Data exclusions	No data were excluded
Replication	Each experiment was replicated at least two to three times on different dates. All attempts at replication were successful
Randomization	No statistical methods were used to predetermine sample size. All mice were included in the analyses. Mice were randomly allocated to the different groups on the basis of cage, genotype, and litter size. For all experiments, we aimed to have the same number of mice in the control and experimental groups.
Blinding	The readout of our in vivo experiments was the body temperature recorded using an intrarectal probe; in consideration that the registration of temperature data does not require of the investigator evaluation, thus no blinded recorded was required

Behavioural & social sciences study design

All studies must disclose on these points even when the disclosure is negative.

Study description	N/A
Research sample	
Sampling strategy	
Data collection	
Timing	
Data exclusions	
Non-participation	
Randomization	

Ecological, evolutionary & environmental sciences study design

All studies must disclose on these points even when the disclosure is negative.

Study description	N/A
Research sample	
Sampling strategy	
Data collection	
Timing and spatial scale	
Data exclusions	
Reproducibility	
Randomization	
Blinding	

Did the study involve field work? ☐ Yes ☐ No

Field work, collection and transport

Field conditions	N/A
Location	
Access & import/export	
Disturbance	

Reporting for specific materials, systems and methods

We require information from authors about some types of materials, experimental systems and methods used in many studies. Here, indicate whether each material, system or method listed is relevant to your study. If you are not sure if a list item applies to your research, read the appropriate section before selecting a response.

Materials & experimental systems

n/a	Involved in the study	
☐	☑	Antibodies
☐	☑	Eukaryotic cell lines
☑	☐	Palaeontology and archaeology
☐	☑	Animals and other organisms
☑	☐	Clinical data
☑	☐	Dual use research of concern
☑	☐	Plants

Methods

n/a	Involved in the study	
☑	☐	ChIP-seq
☐	☑	Flow cytometry
☑	☐	MRI-based neuroimaging

Antibodies

Antibodies used

For flow cytometry:

CD63-APC, Biolegend, catalog number 143906

CD117-PE-Cy7, Biolegend, catalog number 105814

FcεRI-PE, Biolegend, catalog number 134308

CD45-Percp-Cy5.5, Biolegend, catalog number 103132

Avidin-FITC, BD Pharmingen, catalog number 554057

For imaging:

α-mouse CD63 (MBL, Rat IgG 1:100, Cat # D263-3)
α-mouse ASC (EMD Millipore, mouse IgG, 1:1000, Cat # 04-147)
α-mouse NLRP3 (Novus Biological, rabbit IgG 1: 50, Cat # NBP2-12446)
α-mouse α-tubulin (Sigma Aldrich, mouse IgG, 1:250, Cat # T5168)
α-mouse α-dynein (Sigma Aldrich, mouse IgG, 1: 250, Cat # MAB1618)
α-mouse IL-1β (Abcam, rabbit IgG, 1:100, Cat # ab9722)
goat α-rat IgG AF488 (Thermo Scientific, Cat # A-11006)
chicken α-rabbit IgG AF568 (Abcam, Cat # ab175470)
donkey α-mouse IgG AF647 (Abcam, Cat # ab6706)
donkey α-mouse IgG AF568 (Abcam, Cat # ab175472)
chicken α-mouse IgG AF568 (Abcam, Cat # ab175473)

For proteins studies:

α-CD63 (Santa Cruz, Cat # sc-5275)
α-NLRP3 (Novus Biologicals, Cat # NBP2-12446))
α-ASC (Santa Cruz, sc-514414)
α- NEK7 (Novus Biological, Cat # NBP-31110)
α-GFP-CD63 (10 µg, SinoBiological, Cat # MG50557-ANG)
α-Flag-NLRP3/-PYD/-NBD/-LRR (10 µg each, Addgene, Cat # 75137, 75140, 75141)
α-Flag-ASC/-PYD/-CARD truncate (10 µg each, Addgene, 75134)
α-Flag (Cell Signalling, Cat # 8146)
α-GFP (Santa Cruz, Cat # sc-9996)
α-Myc (Cell Signalling, Cat # 5605)
α-Syk (Cell Signaling, Cat # 13198)
α-pSyk (Tyr525/526) Cell Signaling, Cat # 2710)
α-PLCγ (Cell Signaling, Cat # 2822)
α-pPLCγ (Cell Signaling, Cat # 14008)
α-ERK1/2 (Cell Signaling, Cat # 4695)
α-pERK1/2 (Cell Signaling, Cat # 4377),
α-p38 (Cell Signaling, Cat # 8690)
α-p-p38 (Cell Signaling, Cat # 4511)
α-JNK (Cell Signaling, Cat # 9252)
α-pJNK (Cell Signaling, Cat # 9255)
α-Pyk2 (Cell Signaling, Cat # 3292S)
α-pPyk2 (Cell Signaling, Cat # 3291)
α-NEK7 (Novus Biological, Cat # NBP-31110)
α-pASC (ECM, AP5631, Cat # AP5631)

Validation

All flow cytometry used antibodies were validated by comparing their staining pattern on BMMCs during different timing of differentiation by FACS. For imaging experiments the staining validation was confirmed using the secondary antibodies alone. The exception were NLRP3 and ACS antibodies which we tested specificity by confirming lack of signal in Nlrp3-/-, and Asc-/- BMMCs.

Eukaryotic cell lines

Policy information about cell lines and Sex and Gender in Research

Cell line source(s)	RBL-2H3 and HEK293T cells were purchased from ATCC. LAD2 cells were a kind gift from Dr. A.S AS Kirshenbaum at the NIH
Authentication	We verified RBL-2H3 and LAD2 purity by staining microscopy for MCs granule components, and cell surface markers, FceR1 and Kit receptors by FACs.
Mycoplasma contamination	n/a
Commonly misidentified lines (See ICLAC register)	

Palaeontology and Archaeology

Specimen provenance	N/A
Specimen deposition	
Dating methods	

☐ Tick this box to confirm that the raw and calibrated dates are available in the paper or in Supplementary Information.

Ethics oversight	

Note that full information on the approval of the study protocol must also be provided in the manuscript.

Animals and other research organisms

Policy information about studies involving animals; ARRIVE guidelines recommended for reporting animal research, and Sex and Gender in Research

Laboratory animals	MC-deficient mice (*Wsh/Wsh*; '*Sash*') were purchased from Jackson Laboratories and bred in-house at Duke-NUS Medical School vivarium, Singapore. Germ-free mice on the C57BL/6 background were purchased from either Biological Resource Center (BRC), Agency for Science, Technology, and Research (A*STAR), Singapore)/In Vivos (Singapore), and maintained in a germ-free facility. *Nlrp3*$^{-/-}$ were procured from the University of Lausanne (Switzerland) and *Asc*$^{-/-}$ and *Caspase-1*$^{-/-}$ from Genetech (USA) and were maintained at BRC (A*STAR), Singapore, under specific pathogen-free conditions.
Wild animals	No wild animals were involved in our study.
Reporting on sex	N/A
Field-collected samples	No field-collected samples were involved in our study.
Ethics oversight	All animal experiments were performed in compliance with the guidelines from the national care and use of laboratory animals and were approved by the Institutional Animal Care and Use Committee (IACUC), SingHealth (Protocol # 2015/SHS/1121), and BRC, A*STAR (Protocol #161113), Singapore

Note that full information on the approval of the study protocol must also be provided in the manuscript.

Clinical data

Policy information about clinical studies

All manuscripts should comply with the ICMJE guidelines for publication of clinical research and a completed CONSORT checklist must be included with all submissions.

Clinical trial registration	N/A
Study protocol	
Data collection	
Outcomes	

Dual use research of concern

Policy information about dual use research of concern

Hazards

Could the accidental, deliberate or reckless misuse of agents or technologies generated in the work, or the application of information presented in the manuscript, pose a threat to:

No	Yes	
☑	☐	Public health
☑	☐	National security
☑	☐	Crops and/or livestock
☑	☐	Ecosystems
☑	☐	Any other significant area

Experiments of concern

Does the work involve any of these experiments of concern:

No	Yes	
☑	☐	Demonstrate how to render a vaccine ineffective
☑	☐	Confer resistance to therapeutically useful antibiotics or antiviral agents
☑	☐	Enhance the virulence of a pathogen or render a nonpathogen virulent
☑	☐	Increase transmissibility of a pathogen
☑	☐	Alter the host range of a pathogen
☑	☐	Enable evasion of diagnostic/detection modalities
☑	☐	Enable the weaponization of a biological agent or toxin
☑	☐	Any other potentially harmful combination of experiments and agents

Plants

Seed stocks	N/A
Novel plant genotypes	
Authentication	

ChIP-seq

Data deposition

☐ Confirm that both raw and final processed data have been deposited in a public database such as GEO.

☐ Confirm that you have deposited or provided access to graph files (e.g. BED files) for the called peaks.

Data access links<br>*May remain private before publication.*	N/A
Files in database submission	
Genome browser session<br>(e.g. UCSC)	

Methodology

Replicates	
Sequencing depth	
Antibodies	
Peak calling parameters	
Data quality	

Software

Flow Cytometry

Plots

Confirm that:

☑ The axis labels state the marker and fluorochrome used (e.g. CD4-FITC).

☑ The axis scales are clearly visible. Include numbers along axes only for bottom left plot of group (a 'group' is an analysis of identical markers).

☑ All plots are contour plots with outliers or pseudocolor plots.

☑ A numerical value for number of cells or percentage (with statistics) is provided.

Methodology

Sample preparation	BMMCs were stained in PEB buffer (2 mM EDTA and 0.5% bovine serum albumin in PBS). Cells were stained under dark for 30 min in PEB buffer containing antibodies, washed thrice with ice cold PBS.
Instrument	BD LSR II (BD Biosciences) instrument
Software	FACSDiva software (BD Biosciences) for data collection and FlowJo (Tree Star) for data analysis.
Cell population abundance	250000-500000 BMMCs were stained for each condition indicated
Gating strategy	In all experiments, debris were excluded by using Forward scatter/Side scatter (FSC/SSC). Doublets were excluded by double forward (FSC-A and FSC-W), and side scatter (SSC-A and SSC-H). CD45+CD117+ FcεRI+ cells were analyzed for the expression of CD63

☐ Tick this box to confirm that a figure exemplifying the gating strategy is provided in the Supplementary Information.

Magnetic resonance imaging

Experimental design

Design type	N/A
Design specifications	
Behavioral performance measures	
Imaging type(s)	
Field strength	
Sequence & imaging parameters	
Area of acquisition	

Diffusion MRI ☐ Used ☐ Not used

Preprocessing

Preprocessing software	
Normalization	
Normalization template	
Noise and artifact removal	
Volume censoring	

Statistical modeling & inference

Model type and settings	
Effect(s) tested	

Specify type of analysis: ☐ Whole brain ☐ ROI-based ☐ Both

Statistic type for inference

(See Eklund et al. 2016)

Correction

Models & analysis

n/a	Involved in the study
☐	☐ Functional and/or effective connectivity
☐	☐ Graph analysis
☐	☐ Multivariate modeling or predictive analysis

Functional and/or effective connectivity

Graph analysis

Multivariate modeling and predictive analysis

This checklist template is licensed under a Creative Commons Attribution 4.0 International License, which permits use, sharing, adaptation, distribution and reproduction in any medium or format, as long as you give appropriate credit to the original author(s) and the source, provide a link to the Creative Commons license, and indicate if changes were made. The images or other third party material in this article are included in the article's Creative Commons license, unless indicated otherwise in a credit line to the material. If material is not included in the article's Creative Commons license and your intended use is not permitted by statutory regulation or exceeds the permitted use, you will need to obtain permission directly from the copyright holder. To view a copy of this license, visit http://creativecommons.org/licenses/by/4.0/
